# Supplementary material for: Principles of mitoribosomal small subunit assembly in eukaryotes
Source: Nature. 2022 Dec 8;614(7946):175–81. doi: 10.1038/s41586-022-05621-0 (PMC9892005; doi:10.1038/s41586-022-05621-0)
Supplement: Supplementary file 1 — Supplementary Figs. 1–27. [file 41586_2022_5621_MOESM1_ESM.pdf]

nature portfolio

https://doi.org/10.1038/s41586-022-05621-0

Supplementary information

Principles of mitoribosomal small subunit assembly in eukaryotes

In the format provided by the authors and unedited

Supplementary Information

Principles of mitoribosomal small subunit assembly in eukaryotes

Nathan J. Harper[1,2,3], Chloe Burnside[1,2,3], and Sebastian Klinge[1,*]

[1] Laboratory of Protein and Nucleic Acid Chemistry, The Rockefeller University, New York, New York 10065, USA

[2] Tri-Institutional Training Program in Chemical Biology, The Rockefeller University, New York, New York 10065, USA

[3] These authors contributed equally to this work

[*] Correspondence: klinge@rockefeller.edu (S.K.)

TABLE OF CONTENTS

Supplementary Fig. 1 - Uncropped gel images.

Supplementary Fig. 2 - Biallelic editing of human cell lines and human complex purification.

Supplementary Fig. 3 - Human METTL17 dataset cryo-EM data collection and analysis.

Supplementary Fig. 4 - Cryo-EM processing of human State A.

Supplementary Fig. 5 - Cryo-EM processing of human State B.

Supplementary Fig. 6 - Cryo-EM processing of human State C.

Supplementary Fig. 7 - Cryo-EM processing of human State D.

Supplementary Fig. 8 - Cryo-EM processing of human State E.

Supplementary Fig. 9 - Cryo-EM processing of human State C*.

Supplementary Fig. 10 - Local resolution analysis of human State A.

Supplementary Fig. 11 - Local resolution analysis of human State B.

Supplementary Fig. 12 - Local resolution analysis of human State C.

Supplementary Fig. 13 - Local resolution analysis of human State D.

Supplementary Fig. 14 - Local resolution analysis of human State E.

Supplementary Fig. 15 - Local resolution analysis of human State C*.

Supplementary Fig. 16 - Validation of composite maps for human States A and B.

Supplementary Fig. 17 - Validation of composite maps for human States C and D.

Supplementary Fig. 18 - Validation of composite maps for human States E and C*.

Supplementary Fig. 19 - Representative cryo-EM densities and models for human structures.

Supplementary Fig. 20 - Purification of yeast mtSSU assembly intermediates.

Supplementary Fig. 21 - Initial cryo-EM processing of yeast Ccm1-containing particles.

Supplementary Fig. 22 - Cryo-EM processing of yeast States 1 and 2.

Supplementary Fig. 23 - Cryo-EM processing of yeast Rsm22-containing particles (State 3).

Supplementary Fig. 24 - Local resolution analysis of yeast reconstructions

Supplementary Fig. 25 - Validation of composite maps for yeast States 1-3.

Supplementary Fig. 26 - Representative cryo-EM densities and models for yeast structures.

Supplementary Fig. 27 - Pet127 knockout results in accumulation of pre-15S rRNA and a decrease in bound assembly factors.

Supplementary References

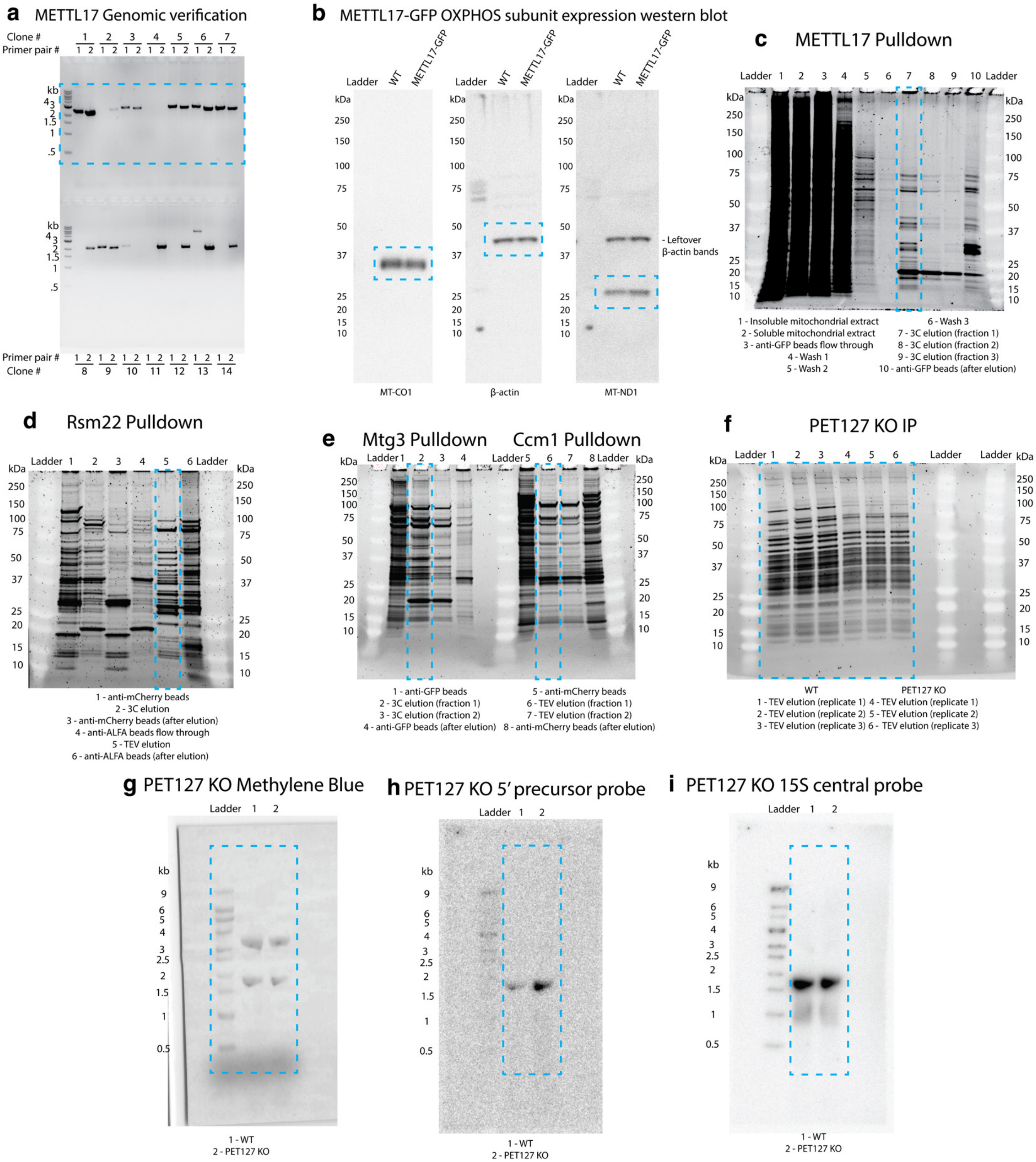
a METTL17 Genomic verification
Clone # 1 2 3 4 5 6 7
Primer pair # 1 2 1 2 1 2 1 2 1 2 1 2 1 2
kb 4 3 2 1.5 1 .5
Primer pair # 1 2 1 2 1 2 1 2 1 2 1 2 1 2
Clone # 8 9 10 11 12 13 14
b METTL17-GFP OXPHOS subunit expression western blot
Ladder WT METTL17-GFP
kDa 250 150 100 75 50 37 25 20 15 10
MT-CO1
β-actin
MT-ND1
- Leftover β-actin bands
c METTL17 Pulldown
Ladder 1 2 3 4 5 6 7 8 9 10 Ladder
1 - Insoluble mitochondrial extract
2 - Soluble mitochondrial extract
3 - anti-GFP beads flow through
4 - Wash 1
5 - Wash 2
6 - Wash 3
7 - 3C elution (fraction 1)
8 - 3C elution (fraction 2)
9 - 3C elution (fraction 3)
10 - anti-GFP beads (after elution)
d Rsm22 Pulldown
Ladder 1 2 3 4 5 6 Ladder
1 - anti-mCherry beads
2 - 3C elution
3 - anti-mCherry beads (after elution)
4 - anti-ALFA beads flow through
5 - TEV elution
6 - anti-ALFA beads (after elution)
e Mtg3 Pulldown Ccm1 Pulldown
Ladder 1 2 3 4 Ladder 5 6 7 8 Ladder
1 - anti-GFP beads
2 - 3C elution (fraction 1)
3 - 3C elution (fraction 2)
4 - anti-GFP beads (after elution)
5 - anti-mCherry beads
6 - TEV elution (fraction 1)
7 - TEV elution (fraction 2)
8 - anti-mCherry beads (after elution)
f PET127 KO IP
Ladder 1 2 3 4 5 6 Ladder Ladder
WT
1 - TEV elution (replicate 1)
2 - TEV elution (replicate 2)
3 - TEV elution (replicate 3)
PET127 KO
4 - TEV elution (replicate 1)
5 - TEV elution (replicate 2)
6 - TEV elution (replicate 3)
g PET127 KO Methylene Blue
h PET127 KO 5′ precursor probe
i PET127 KO 15S central probe
Ladder 1 2
kb 9 6 5 4 3 2.5 2 1.5 1 0.5
1 - WT
2 - PET127 KO

Supplementary Fig. 1. Uncropped gel images.

Uncropped gel images relating to: (**a**) Supplementary Fig. 2c, (**b**) Supplementary Fig. 2f, (**c**) Supplementary Fig. 2e, (**d**) Supplementary Fig. 20f, (**e**) Supplementary Fig. 20b,j, (**f**) Supplementary Fig. 27c, (**g-i**) Supplementary Fig. 27b. Blue dotted rectangles show approximate cropping dimensions used in related figures.

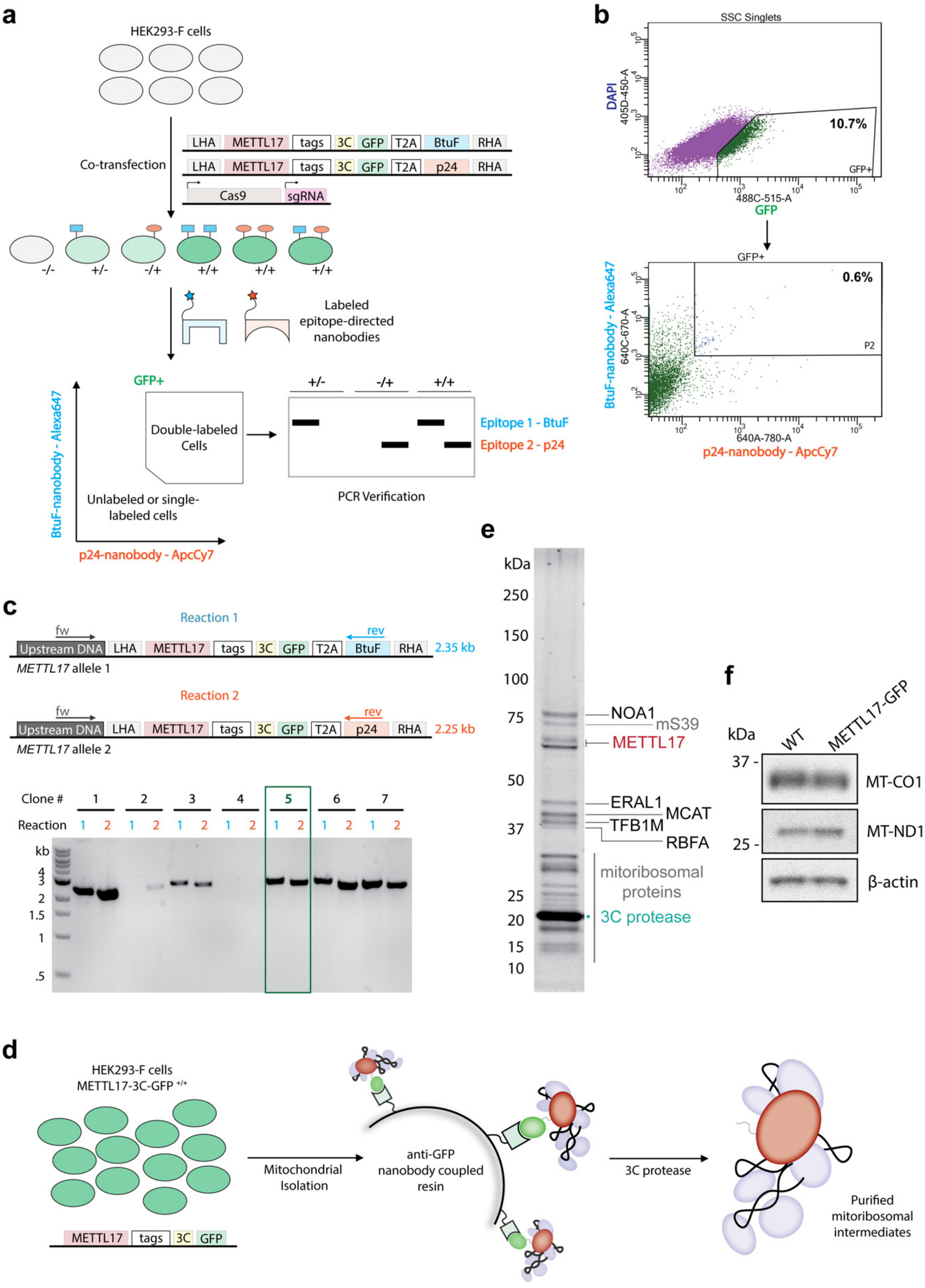
a
HEK293-F cells
Co-transfection
LHA METTL17 tags 3C GFP T2A BtuF RHA
LHA METTL17 tags 3C GFP T2A p24 RHA
Cas9 sgRNA
-/- +/- -/+ +/+ +/+ +/+
Labeled epitope-directed nanobodies
GFP+
Double-labeled Cells
Unlabeled or single-labeled cells
BtuF-nanobody - Alexa647
p24-nanobody - ApcCy7
+/- -/+ +/+
Epitope 1 - BtuF
Epitope 2 - p24
PCR Verification
b
SSC Singlets
DAPI
405D-450-A
10.7%
GFP+
488C-515-A
GFP
GFP+
0.6%
P2
BtuF-nanobody - Alexa647
640C-670-A
640A-780-A
p24-nanobody - ApcCy7
c
Reaction 1
fw
rev
Upstream DNA LHA METTL17 tags 3C GFP T2A BtuF RHA 2.35 kb
METTL17 allele 1
Reaction 2
Upstream DNA LHA METTL17 tags 3C GFP T2A p24 RHA 2.25 kb
METTL17 allele 2
Clone # 1 2 3 4 5 6 7
Reaction 1 2 1 2 1 2 1 2 1 2 1 2 1 2
kb 4 3 2 1.5 1 .5
d
HEK293-F cells METTL17-3C-GFP +/+
METTL17 tags 3C GFP
Mitochondrial Isolation
anti-GFP nanobody coupled resin
3C protease
Purified mitoribosomal intermediates
e
kDa 250 150 100 75 50 37 25 20 15 10
NOA1
mS39
METTL17
ERAL1
MCAT
TFB1M
RBFA
mitoribosomal proteins
3C protease
f
kDa
WT
METTL17-GFP
37 -
25 -
MT-CO1
MT-ND1
β-actin

Supplementary Fig. 2. Biallelic editing of human cell lines and human complex purification.

(**a**) Simplified scheme of genomic editing using SNEAK-PEEC targeting METTL17. See methods for details. (**b**) FACS sorting on cells transfected with repair templates and *Sp*Cas9 targeting METTL17. Inset percentages denote proportion of cells positive for GFP (upper panel), and proportion of GFP positive cells with signal for both cell surface display epitopes (bottom panel). Cells from P2 were sorted to generate single cell clonal populations. (**c**) PCR verification of single cell clones from FACS sorting. Primer annealing locations are shown in genomic context in the upper panel. Samples were run on a 1% Agarose-TAE gel with EtBr. Highlighted clone was used for subsequent experiments. (**d**) Purification scheme for METTL17-containing mitoribosomal intermediates. (**e**) Representative SDS-PAGE gel of eluted sample. Proteins were identified with LC-MS/MS (Supplementary Data S1). Immunoprecipitation experiments were repeated at least 3 times with similar results. (**f**) Western blot showing unchanged expression of mitochondrially encoded OXPHOS subunits MT-CO1 and MT-ND1 in METTL17-GFP tagged cell line versus WT cells. The western blot was performed 3 times with similar results. For gel source data, see Supplementary Fig. 1.

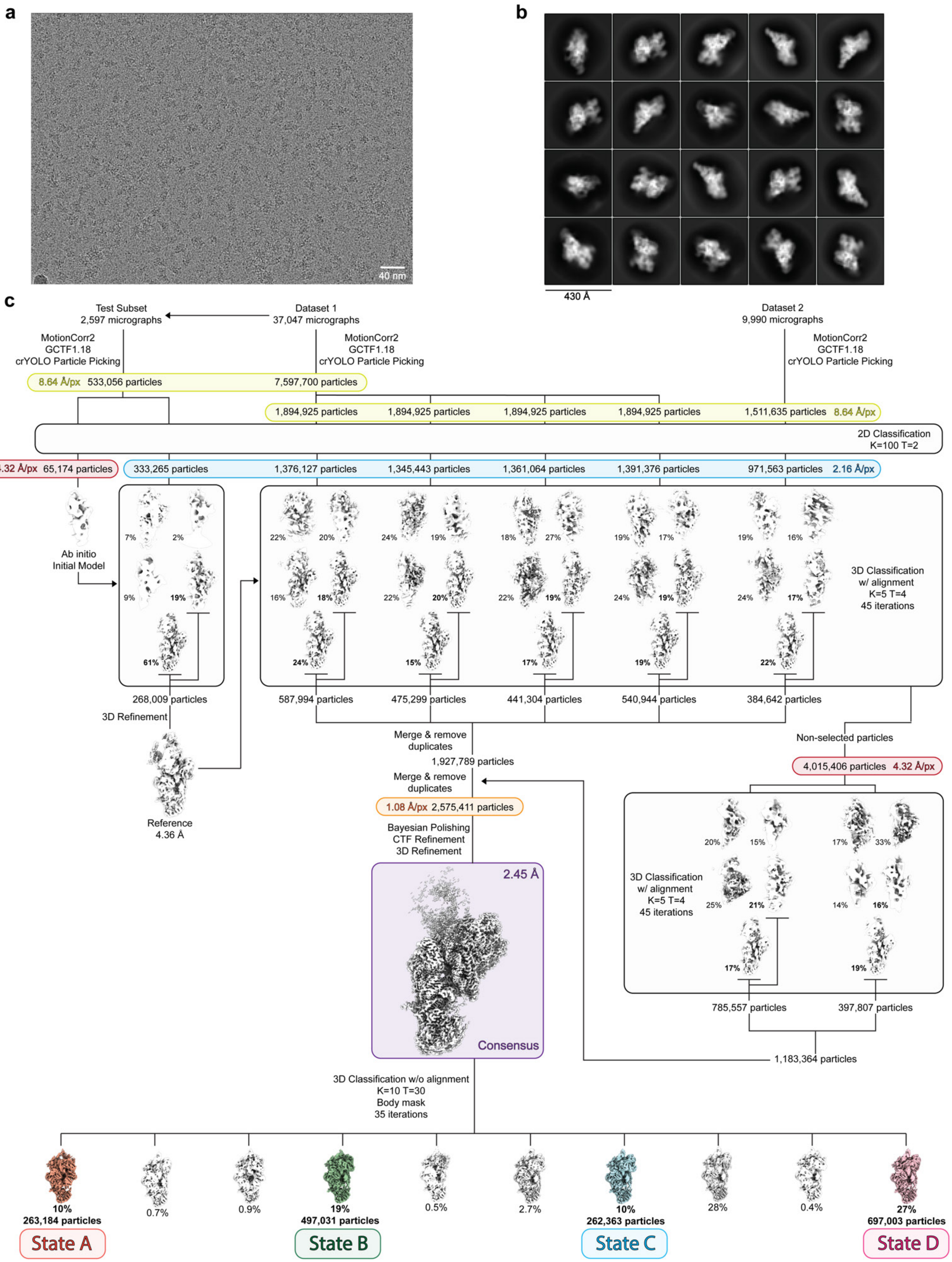
a
40 nm
b
430 Å
c
Test Subset
2,597 micrographs
Dataset 1
37,047 micrographs
Dataset 2
9,990 micrographs
MotionCorr2
GCTF1.18
crYOLO Particle Picking
8.64 Å/px 533,056 particles
7,597,700 particles
1,894,925 particles
1,894,925 particles
1,894,925 particles
1,894,925 particles
1,511,635 particles 8.64 Å/px
2D Classification
K=100 T=2
4.32 Å/px 65,174 particles
333,265 particles
1,376,127 particles
1,345,443 particles
1,361,064 particles
1,391,376 particles
971,563 particles 2.16 Å/px
Ab initio
Initial Model
7% 2% 9% 19% 61%
22% 20% 16% 18% 24%
24% 19% 22% 20% 15%
18% 27% 22% 19% 17%
19% 17% 24% 19% 19%
19% 16% 24% 17% 22%
3D Classification
w/ alignment
K=5 T=4
45 iterations
268,009 particles
3D Refinement
Reference
4.36 Å
587,994 particles
475,299 particles
441,304 particles
540,944 particles
384,642 particles
Merge & remove
duplicates
1,927,789 particles
Merge & remove
duplicates
1.08 Å/px 2,575,411 particles
Bayesian Polishing
CTF Refinement
3D Refinement
2.45 Å
Consensus
Non-selected particles
4,015,406 particles 4.32 Å/px
20% 15% 25% 21% 17%
17% 33% 14% 16% 19%
3D Classification
w/ alignment
K=5 T=4
45 iterations
785,557 particles
397,807 particles
1,183,364 particles
3D Classification w/o alignment
K=10 T=30
Body mask
35 iterations
10%
263,184 particles
0.7%
0.9%
19%
497,031 particles
0.5%
2.7%
10%
262,363 particles
28%
0.4%
27%
697,003 particles
State A
State B
State C
State D

Supplementary Fig. 3. Human METTL17 dataset cryo-EM data collection and analysis.

(**a**) Motion corrected cryo-EM micrograph representative of 47,037 total micrographs. (**b**) 15 most populated 2D class averages (8x binned, 8.64 Å/px). (**c**) Initial data processing workflow and identification of 4 discrete assembly states.

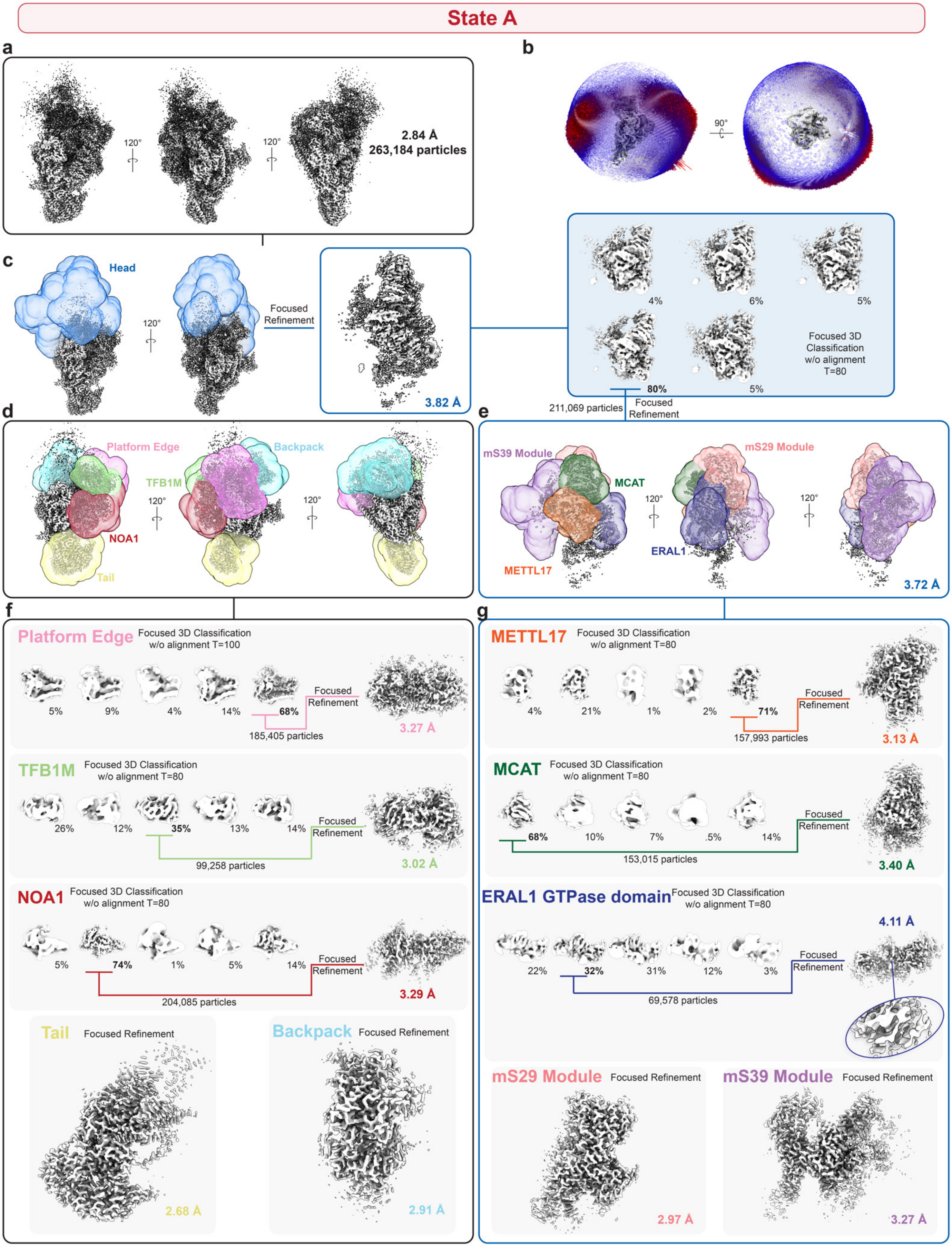

State A
a
120°
120°
2.84 Å
263,184 particles
b
90°
c
Head
120°
Focused Refinement
3.82 Å
4%
6%
5%
80%
5%
Focused 3D Classification w/o alignment T=80
211,069 particles
Focused Refinement
d
Platform Edge
Backpack
TFB1M
120°
120°
NOA1
Tail
e
mS39 Module
mS29 Module
MCAT
120°
120°
ERAL1
METTL17
3.72 Å
f
Platform Edge
Focused 3D Classification w/o alignment T=100
5%
9%
4%
14%
68%
Focused Refinement
185,405 particles
3.27 Å
TFB1M
Focused 3D Classification w/o alignment T=80
26%
12%
35%
13%
14%
Focused Refinement
99,258 particles
3.02 Å
NOA1
Focused 3D Classification w/o alignment T=80
5%
74%
1%
5%
14%
Focused Refinement
204,085 particles
3.29 Å
Tail
Focused Refinement
2.68 Å
Backpack
Focused Refinement
2.91 Å
g
METTL17
Focused 3D Classification w/o alignment T=80
4%
21%
1%
2%
71%
Focused Refinement
157,993 particles
3.13 Å
MCAT
Focused 3D Classification w/o alignment T=80
68%
10%
7%
.5%
14%
Focused Refinement
153,015 particles
3.40 Å
ERAL1 GTPase domain
Focused 3D Classification w/o alignment T=80
4.11 Å
22%
32%
31%
12%
3%
Focused Refinement
69,578 particles
mS29 Module
Focused Refinement
2.97 Å
mS39 Module
Focused Refinement
3.27 Å

Supplementary Fig. 4. Cryo-EM processing of human State A.

(**a**) Different views of the State A overall map. (**b**) Euler angle distribution of the overall refinement. (**c**) Focused refinement and classification of the head domain. (**d**) Masks used in focused classification and refinement for modules in the body domain. (**e**) Masks used in focused classification and refinement for modules in the head domain. (**f,g**) Workflows for focused 3D classification and refinements. Reported resolutions (FSC=0.143) from Relion post-processing are reported next to individual focused maps. Masks used are color coded as in panels d and e.

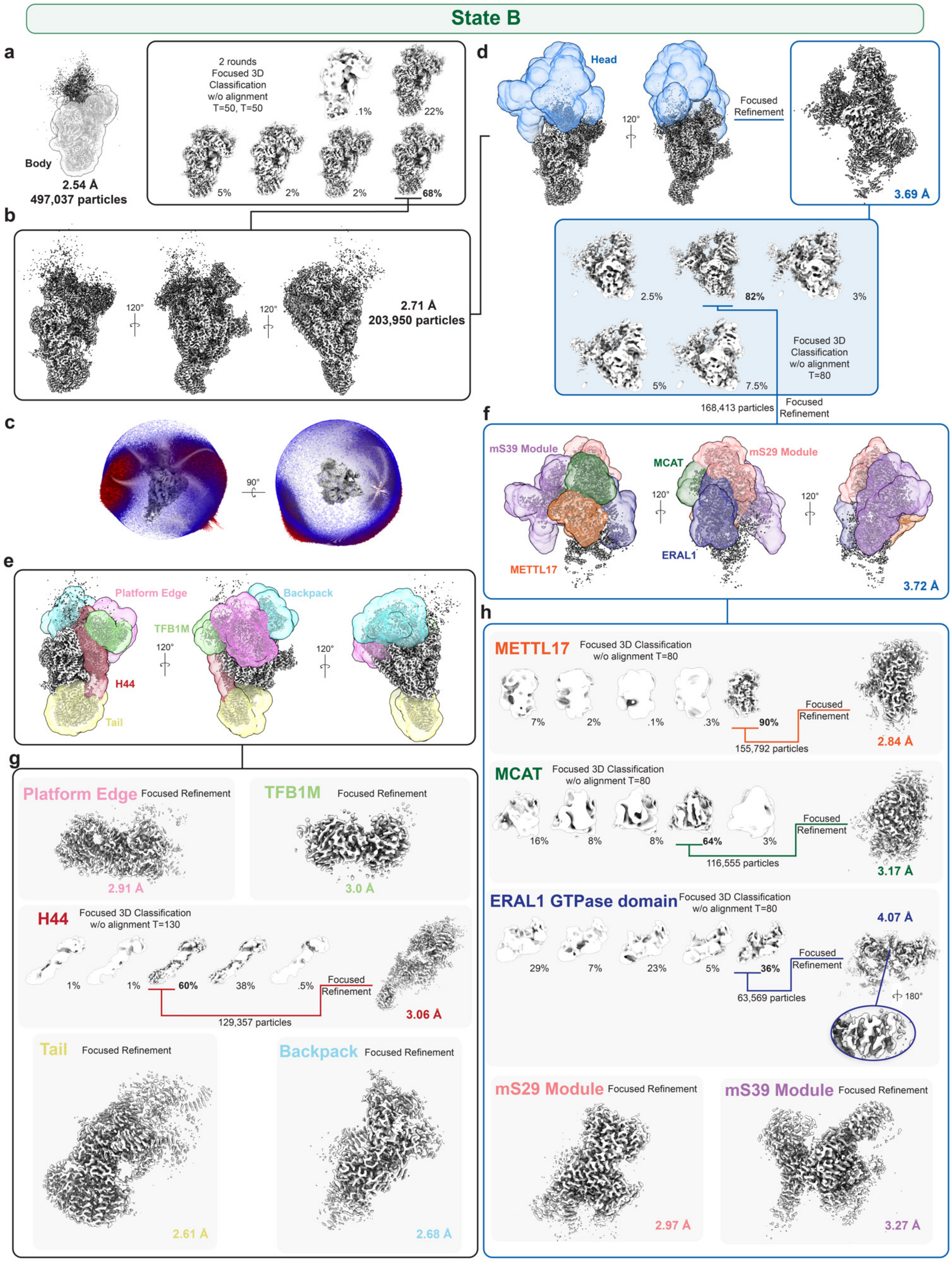
State B
a
Body
2.54 Å
497,037 particles
2 rounds
Focused 3D
Classification
w/o alignment
T=50, T=50
.1%
22%
5%
2%
2%
68%
b
120°
120°
2.71 Å
203,950 particles
c
90°
d
Head
120°
Focused
Refinement
3.69 Å
2.5%
82%
3%
Focused 3D
Classification
w/o alignment
T=80
5%
7.5%
168,413 particles
Focused
Refinement
e
Platform Edge
Backpack
TFB1M
120°
120°
H44
Tail
f
mS39 Module
MCAT
mS29 Module
120°
120°
METTL17
ERAL1
3.72 Å
g
Platform Edge
Focused Refinement
2.91 Å
TFB1M
Focused Refinement
3.0 Å
H44
Focused 3D Classification
w/o alignment T=130
1%
1%
60%
38%
.5%
Focused
Refinement
129,357 particles
3.06 Å
Tail
Focused Refinement
2.61 Å
Backpack
Focused Refinement
2.68 Å
h
METTL17
Focused 3D Classification
w/o alignment T=80
7%
2%
.1%
.3%
90%
Focused
Refinement
155,792 particles
2.84 Å
MCAT
Focused 3D Classification
w/o alignment T=80
16%
8%
8%
64%
3%
Focused
Refinement
116,555 particles
3.17 Å
ERAL1 GTPase domain
Focused 3D Classification
w/o alignment T=80
4.07 Å
29%
7%
23%
5%
36%
Focused
Refinement
63,569 particles
180°
mS29 Module
Focused Refinement
2.97 Å
mS39 Module
Focused Refinement
3.27 Å

Supplementary Fig. 5. Cryo-EM processing of human State B.

(**a**) Initial classification of particles with a body mask to clean the particle stack. (**b**) Different views of the State B overall map. (**c**) Euler angle distribution of the overall refinement. (**d**) Focused refinement and classification of the head domain. (**e**) Masks used in focused classification and refinement for modules in the body domain. (**f**) Masks used in focused classification and refinement for modules in the head domain. (**g,h**) Workflows for focused 3D classification and refinements. Reported resolutions (FSC=0.143) from Relion post-processing are reported next to individual focused maps. Masks used are color coded as in panels e and f.

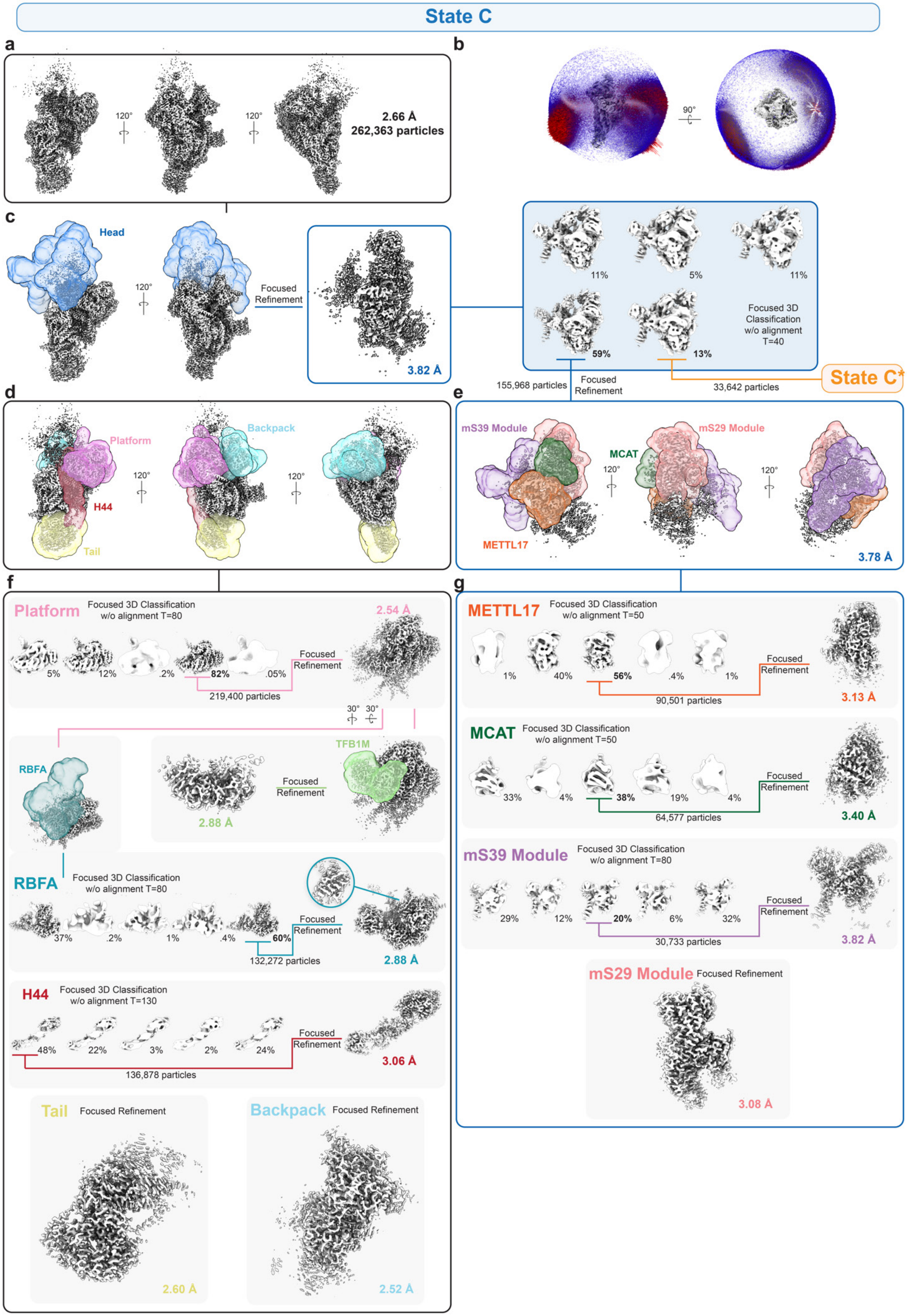

State C
a
120°
120°
2.66 Å
262,363 particles
b
90°
c
Head
120°
Focused Refinement
3.82 Å
11%
5%
11%
59%
13%
Focused 3D Classification w/o alignment T=40
155,968 particles
Focused Refinement
33,642 particles
State C*
d
Platform
Backpack
H44
Tail
120°
120°
e
mS39 Module
mS29 Module
MCAT
METTL17
120°
120°
3.78 Å
f
Platform
Focused 3D Classification w/o alignment T=80
5%
12%
.2%
82%
.05%
Focused Refinement
219,400 particles
2.54 Å
30°
30°
RBFA
TFB1M
Focused Refinement
2.88 Å
RBFA
Focused 3D Classification w/o alignment T=80
37%
.2%
1%
.4%
60%
Focused Refinement
132,272 particles
2.88 Å
H44
Focused 3D Classification w/o alignment T=130
48%
22%
3%
2%
24%
Focused Refinement
136,878 particles
3.06 Å
Tail
Focused Refinement
2.60 Å
Backpack
Focused Refinement
2.52 Å
g
METTL17
Focused 3D Classification w/o alignment T=50
1%
40%
56%
.4%
1%
Focused Refinement
90,501 particles
3.13 Å
MCAT
Focused 3D Classification w/o alignment T=50
33%
4%
38%
19%
4%
Focused Refinement
64,577 particles
3.40 Å
mS39 Module
Focused 3D Classification w/o alignment T=80
29%
12%
20%
6%
32%
Focused Refinement
30,733 particles
3.82 Å
mS29 Module
Focused Refinement
3.08 Å

Supplementary Fig. 6. Cryo-EM processing of human State C.

(**a**) Different views of the State C overall map. (**b**) Euler angle distribution of the overall refinement. (**c**) Focused refinement and classification of the head domain. Particles identified as State C* were further processed independently (Supplementary Fig. 9). (**d**) Masks used in focused classification and refinement for modules in the body domain. (**e**) Masks used in focused classification and refinement for modules in the head domain. (**f,g**) Workflows for focused 3D classification and refinements. Reported resolutions (FSC=0.143) from Relion post-processing are reported next to individual focused maps. Masks used are color coded as in panels d and e.

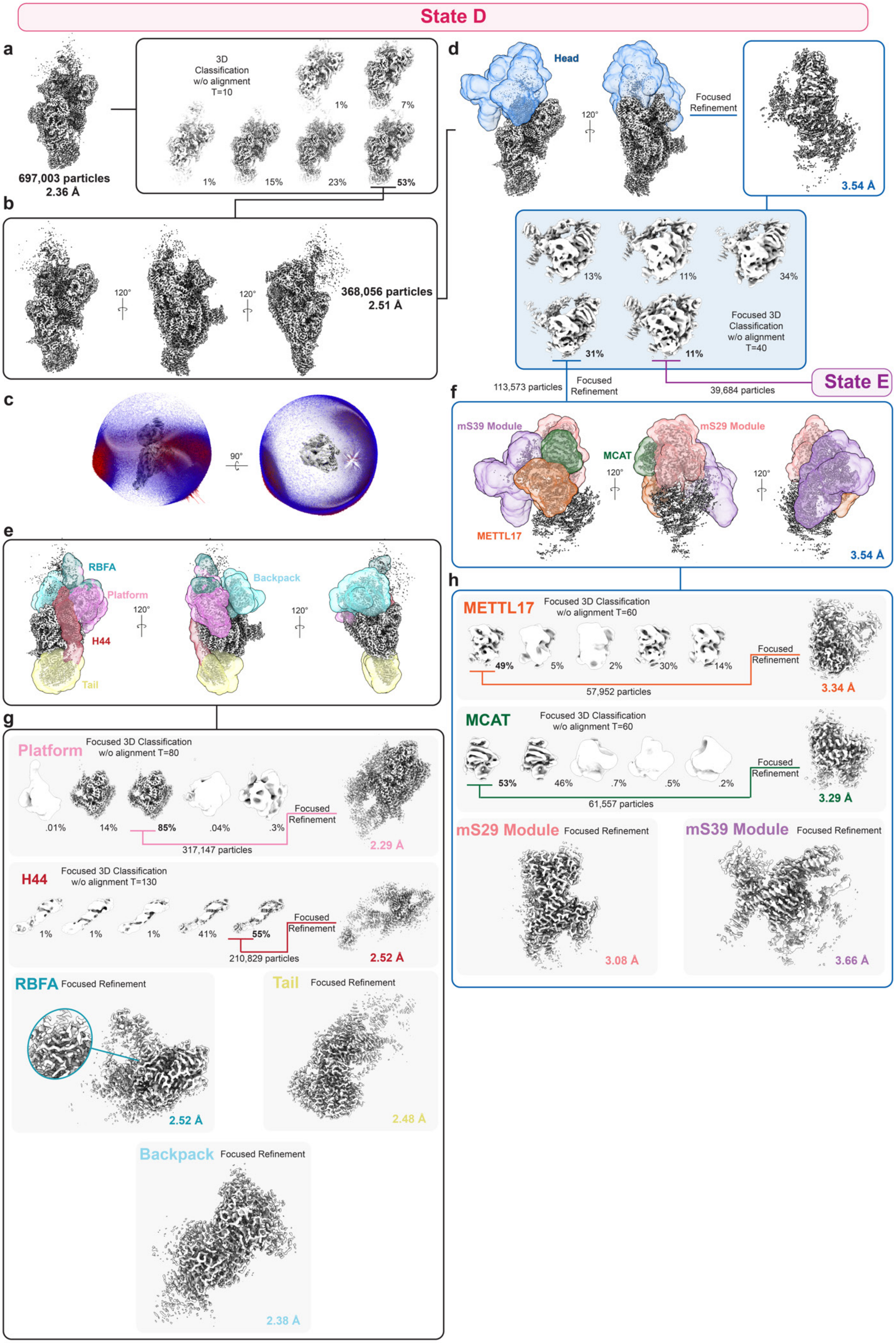
State D
a
3D Classification w/o alignment T=10
1%
7%
1%
15%
23%
53%
697,003 particles
2.36 Å
b
120°
120°
368,056 particles
2.51 Å
c
90°
d
Head
120°
Focused Refinement
3.54 Å
13%
11%
34%
31%
11%
Focused 3D Classification w/o alignment T=40
113,573 particles
Focused Refinement
39,684 particles
State E
e
RBFA
Platform
H44
Tail
Backpack
120°
120°
f
mS39 Module
mS29 Module
MCAT
METTL17
120°
120°
3.54 Å
g
Platform
Focused 3D Classification w/o alignment T=80
.01%
14%
85%
.04%
.3%
Focused Refinement
317,147 particles
2.29 Å
H44
Focused 3D Classification w/o alignment T=130
1%
1%
1%
41%
55%
Focused Refinement
210,829 particles
2.52 Å
RBFA
Focused Refinement
2.52 Å
Tail
Focused Refinement
2.48 Å
Backpack
Focused Refinement
2.38 Å
h
METTL17
Focused 3D Classification w/o alignment T=60
49%
5%
2%
30%
14%
Focused Refinement
57,952 particles
3.34 Å
MCAT
Focused 3D Classification w/o alignment T=60
53%
46%
.7%
.5%
.2%
Focused Refinement
61,557 particles
3.29 Å
mS29 Module
Focused Refinement
3.08 Å
mS39 Module
Focused Refinement
3.66 Å

Supplementary Fig. 7. Cryo-EM processing of human State D.

(**a**) Initial classification of particles without a mask to clean the particle stack. (**b**) Different views of the State D overall map. (**c**) Euler angle distribution of the overall refinement. (**d**) Focused refinement and classification of the head domain. Particles identified as State E were further processed independently (Supplementary Fig. 8). (**e**) Masks used in focused classification and refinement for modules in the body domain. (**f**) Masks used in focused classification and refinement for modules in the head domain. (**g,h**) Workflows for focused 3D classification and refinements. Reported resolutions (FSC=0.143) from Relion post-processing are reported next to individual focused maps. Masks used are color coded as in panels e and f.

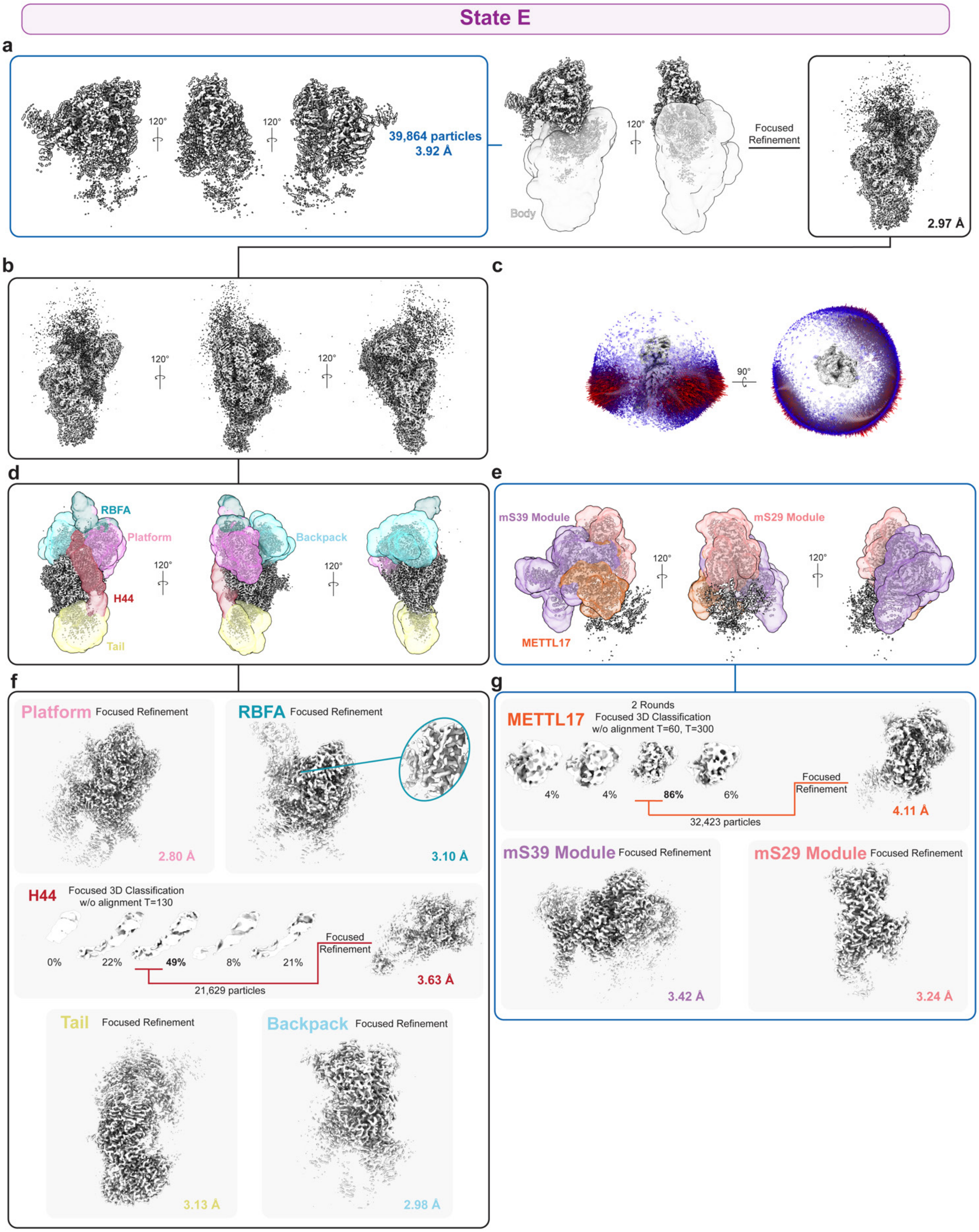

State E
a
120°
120°
39,864 particles
3.92 Å
120°
Body
Focused Refinement
2.97 Å
b
120°
120°
c
90°
d
RBFA
Platform
H44
Tail
120°
Backpack
120°
e
mS39 Module
METTL17
120°
mS29 Module
120°
f
Platform Focused Refinement
2.80 Å
RBFA Focused Refinement
3.10 Å
H44 Focused 3D Classification w/o alignment T=130
0%
22%
49%
8%
21%
Focused Refinement
21,629 particles
3.63 Å
Tail Focused Refinement
3.13 Å
Backpack Focused Refinement
2.98 Å
g
METTL17
2 Rounds
Focused 3D Classification
w/o alignment T=60, T=300
4%
4%
86%
6%
Focused Refinement
32,423 particles
4.11 Å
mS39 Module Focused Refinement
3.42 Å
mS29 Module Focused Refinement
3.24 Å

Supplementary Fig. 8. Cryo-EM processing of human State E.

(**a**) Different views of the State E head map. Particles were then refined using a mask on the body. (**b**) Different views of the body domain. (**c**) Euler angle distribution of the overall (body) refinement. (**d**) Masks used in focused classification and refinement for modules in the body domain. (**e**) Masks used in focused classification and refinement for modules in the head domain. (**f,g**) Workflows for focused 3D classification and refinements. Reported resolutions (FSC=0.143) from Relion post-processing are reported next to individual focused maps. Masks used are color coded as in panels d and e.

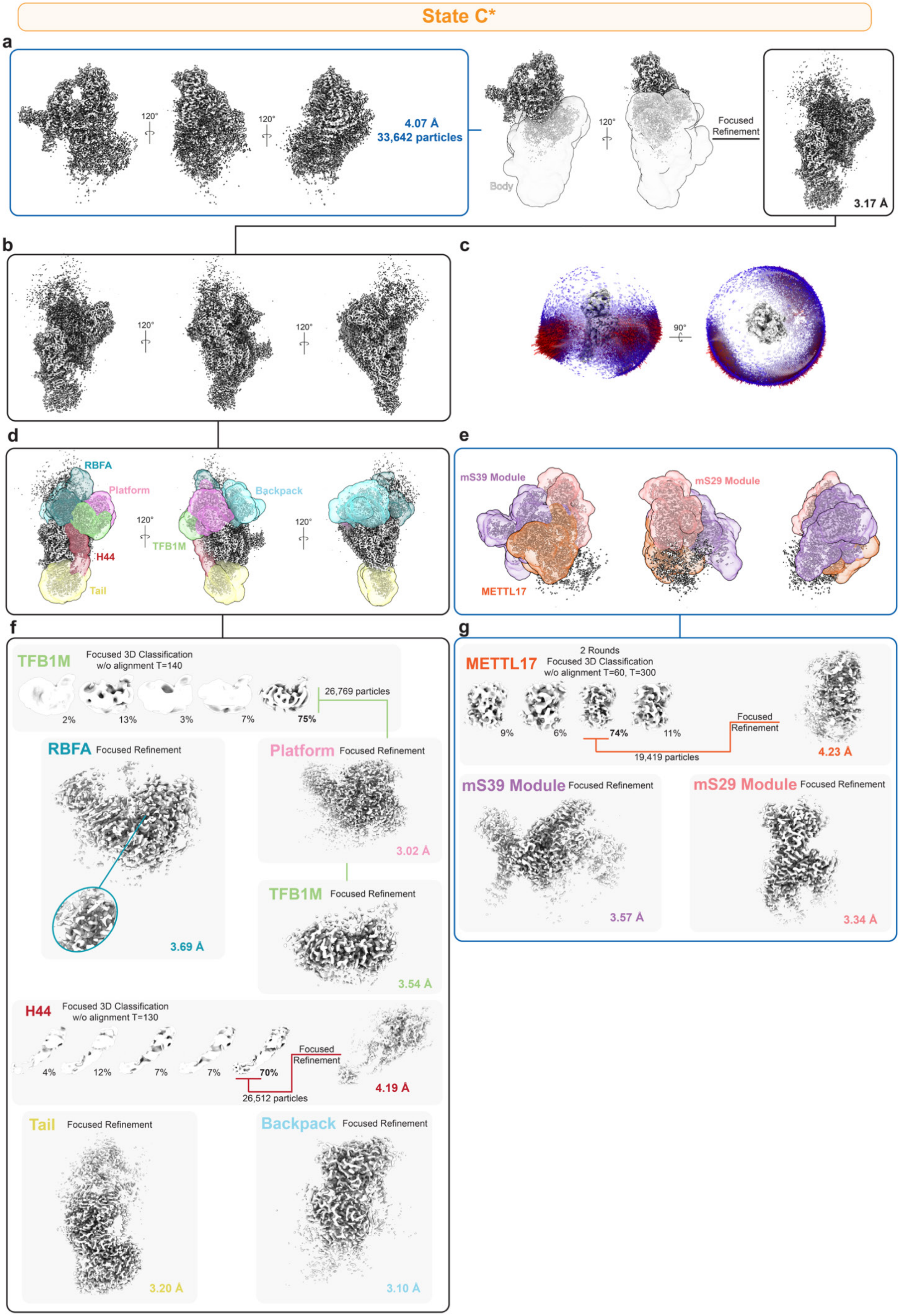

State C*
a
120°
120°
4.07 Å
33,642 particles
Body
120°
Focused Refinement
3.17 Å
b
120°
120°
c
90°
d
RBFA
Platform
Backpack
120°
120°
TFB1M
H44
Tail
e
mS39 Module
mS29 Module
METTL17
f
TFB1M Focused 3D Classification w/o alignment T=140
2%
13%
3%
7%
75%
26,769 particles
RBFA Focused Refinement
3.69 Å
Platform Focused Refinement
3.02 Å
TFB1M Focused Refinement
3.54 Å
H44 Focused 3D Classification w/o alignment T=130
4%
12%
7%
7%
70%
Focused Refinement
26,512 particles
4.19 Å
Tail Focused Refinement
3.20 Å
Backpack Focused Refinement
3.10 Å
g
METTL17 2 Rounds Focused 3D Classification w/o alignment T=60, T=300
9%
6%
74%
11%
Focused Refinement
19,419 particles
4.23 Å
mS39 Module Focused Refinement
3.57 Å
mS29 Module Focused Refinement
3.34 Å

Supplementary Fig. 9. Cryo-EM processing of human State C*.

(**a**) Different views of the State C* head map. Particles were then refined using a mask on the body. (**b**) Different views of the body domain. (**c**) Euler angle distribution of the overall (body) refinement. (**d**) Masks used in focused classification and refinement for modules in the body domain. (**e**) Masks used in focused classification and refinement for modules in the head domain. (**f,g**) Workflows for focused 3D classification and refinements. Reported resolutions (FSC=0.143) from Relion post-processing are reported next to individual focused maps. Masks used are color coded as in panels d and e.

State A

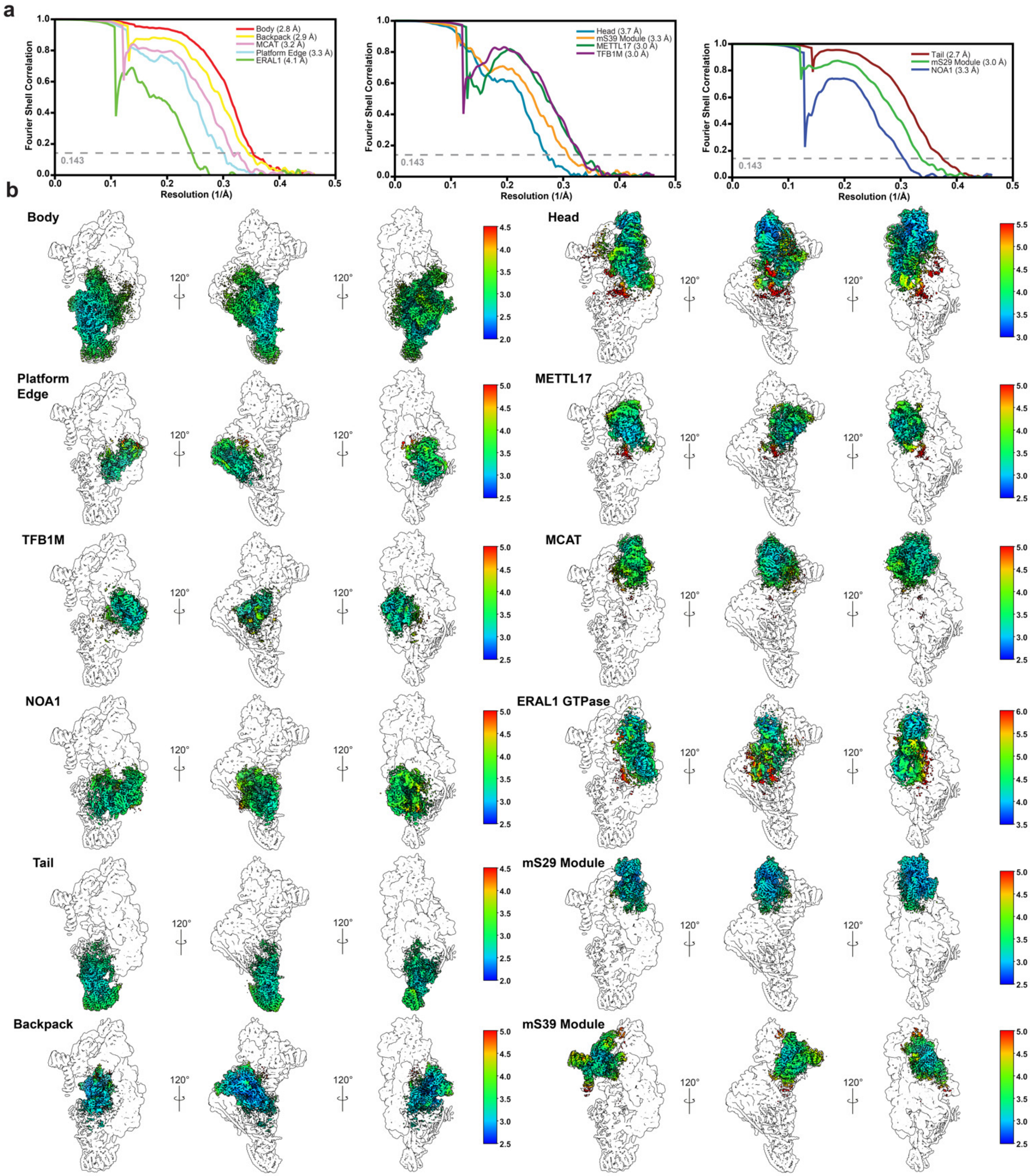
a
Fourier Shell Correlation
Resolution (1/Å)
0.143
Body (2.8 Å)
Backpack (2.9 Å)
MCAT (3.2 Å)
Platform Edge (3.3 Å)
ERAL1 (4.1 Å)
Head (3.7 Å)
mS39 Module (3.3 Å)
METTL17 (3.0 Å)
TFB1M (3.0 Å)
Tail (2.7 Å)
mS29 Module (3.0 Å)
NOA1 (3.3 Å)
b
Body
Head
Platform Edge
METTL17
TFB1M
MCAT
NOA1
ERAL1 GTPase
Tail
mS29 Module
Backpack
mS39 Module
120°

Supplementary Fig. 10. Local resolution analysis of human State A.

(**a**) Solvent-corrected FSC curves for each focused refinement determined at FSC=0.143 via post-processing in Relion. (**b**) Local resolution estimation (calculated in Relion) for each focused map in context with the rest of the reconstruction.

State B

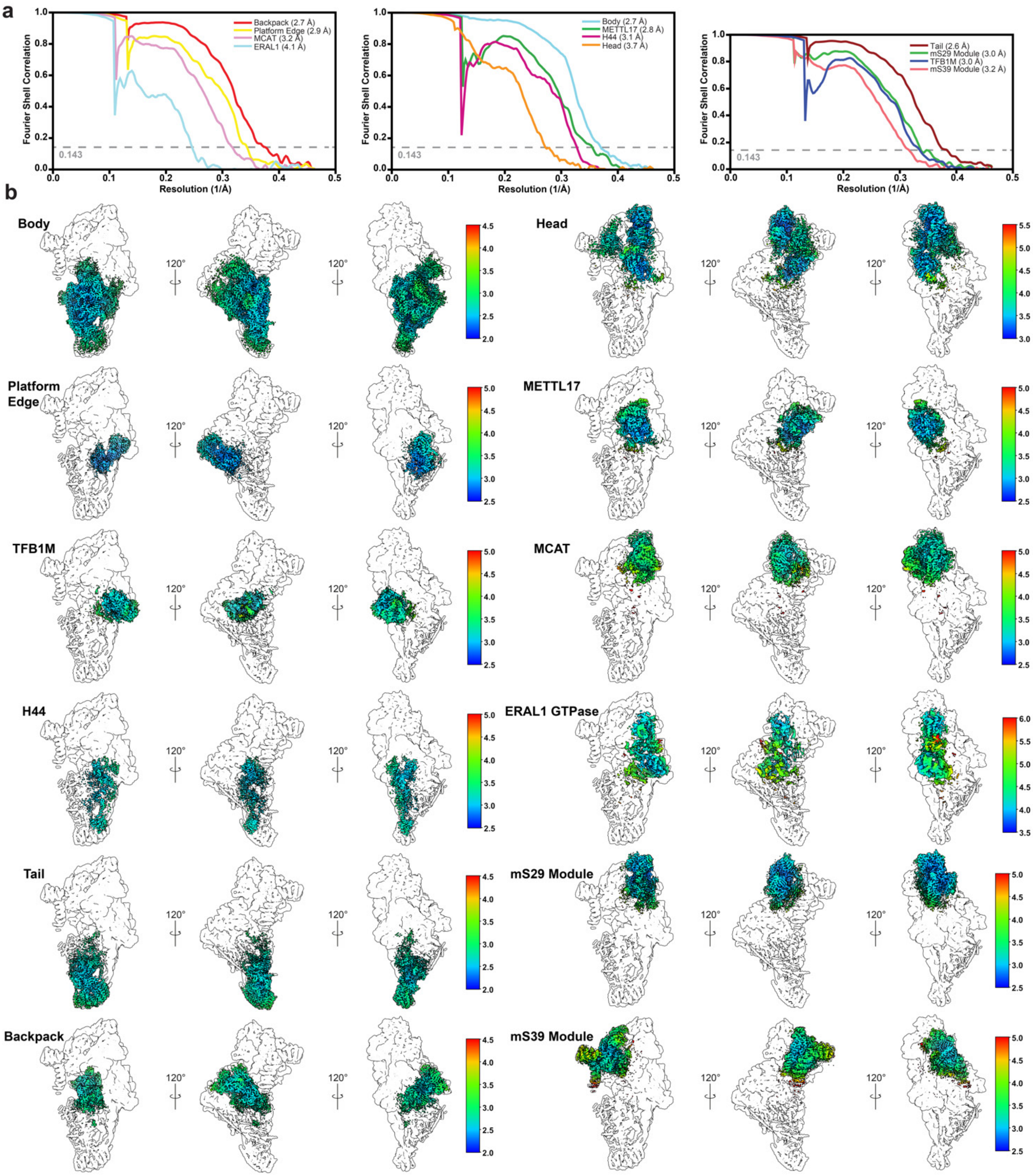
a
Fourier Shell Correlation
Resolution (1/Å)
0.143
Backpack (2.7 Å)
Platform Edge (2.9 Å)
MCAT (3.2 Å)
ERAL1 (4.1 Å)
Body (2.7 Å)
METTL17 (2.8 Å)
H44 (3.1 Å)
Head (3.7 Å)
Tail (2.6 Å)
mS29 Module (3.0 Å)
TFB1M (3.0 Å)
mS39 Module (3.2 Å)
b
Body
Head
Platform Edge
METTL17
TFB1M
MCAT
H44
ERAL1 GTPase
Tail
mS29 Module
Backpack
mS39 Module
120°

Supplementary Fig. 11. Local resolution analysis of human State B.

(**a**) Solvent-corrected FSC curves for each focused refinement determined at FSC=0.143 via post-processing in Relion. (**b**) Local resolution estimation (calculated in Relion) for each focused map in context with the rest of the reconstruction.

State C

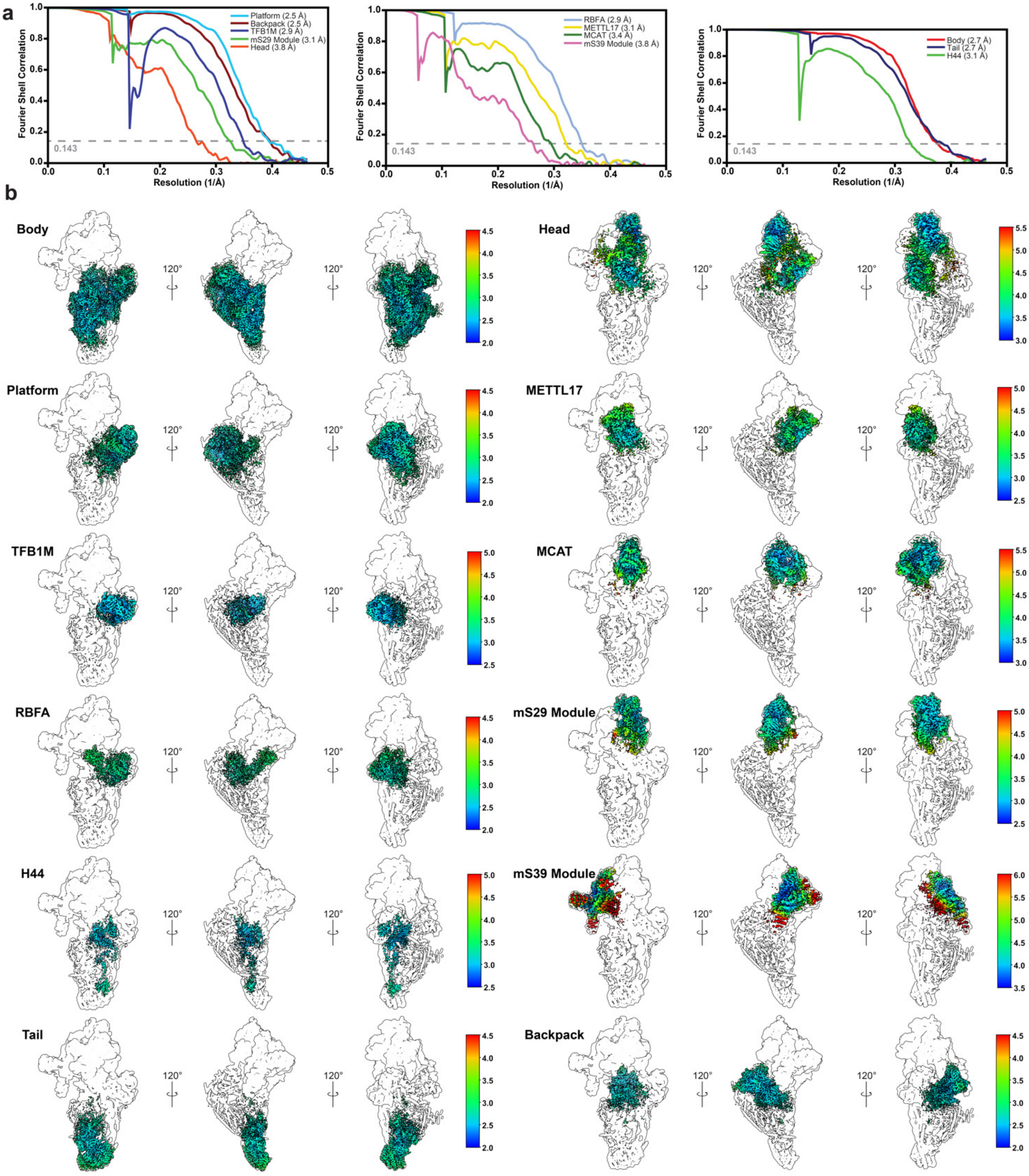

a
Fourier Shell Correlation
Resolution (1/Å)
0.143
Platform (2.5 Å)
Backpack (2.5 Å)
TFB1M (2.9 Å)
mS29 Module (3.1 Å)
Head (3.8 Å)
RBFA (2.9 Å)
METTL17 (3.1 Å)
MCAT (3.4 Å)
mS39 Module (3.8 Å)
Body (2.7 Å)
Tail (2.7 Å)
H44 (3.1 Å)
b
Body
Platform
TFB1M
RBFA
H44
Tail
Head
METTL17
MCAT
mS29 Module
mS39 Module
Backpack
120°

Supplementary Fig. 12. Local resolution analysis of human State C.

(**a**) Solvent-corrected FSC curves for each focused refinement determined at FSC=0.143 via post-processing in Relion. (**b**) Local resolution estimation (calculated in Relion) for each focused map in context with the rest of the reconstruction.

State D

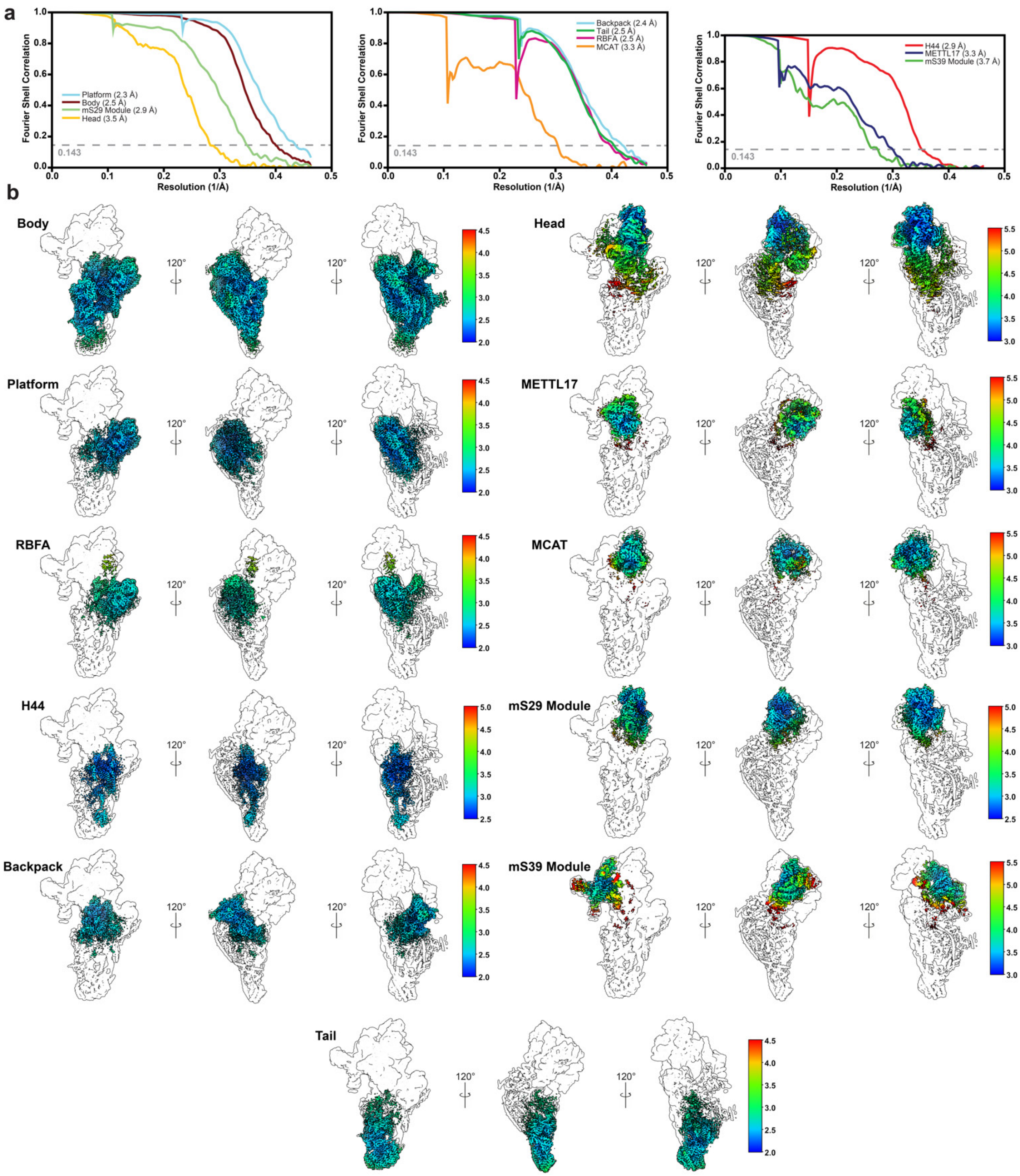
a
Fourier Shell Correlation
Resolution (1/Å)
Platform (2.3 Å)
Body (2.5 Å)
mS29 Module (2.9 Å)
Head (3.5 Å)
0.143
Backpack (2.4 Å)
Tail (2.5 Å)
RBFA (2.5 Å)
MCAT (3.3 Å)
H44 (2.9 Å)
METTL17 (3.3 Å)
mS39 Module (3.7 Å)
b
Body
Head
Platform
METTL17
RBFA
MCAT
H44
mS29 Module
Backpack
mS39 Module
Tail
120°

Supplementary Fig. 13. Local resolution analysis of human State D.

(**a**) Solvent-corrected FSC curves for each focused refinement determined at FSC=0.143 via post-processing in Relion. (**b**) Local resolution estimation (calculated in Relion) for each focused map in context with the rest of the reconstruction.

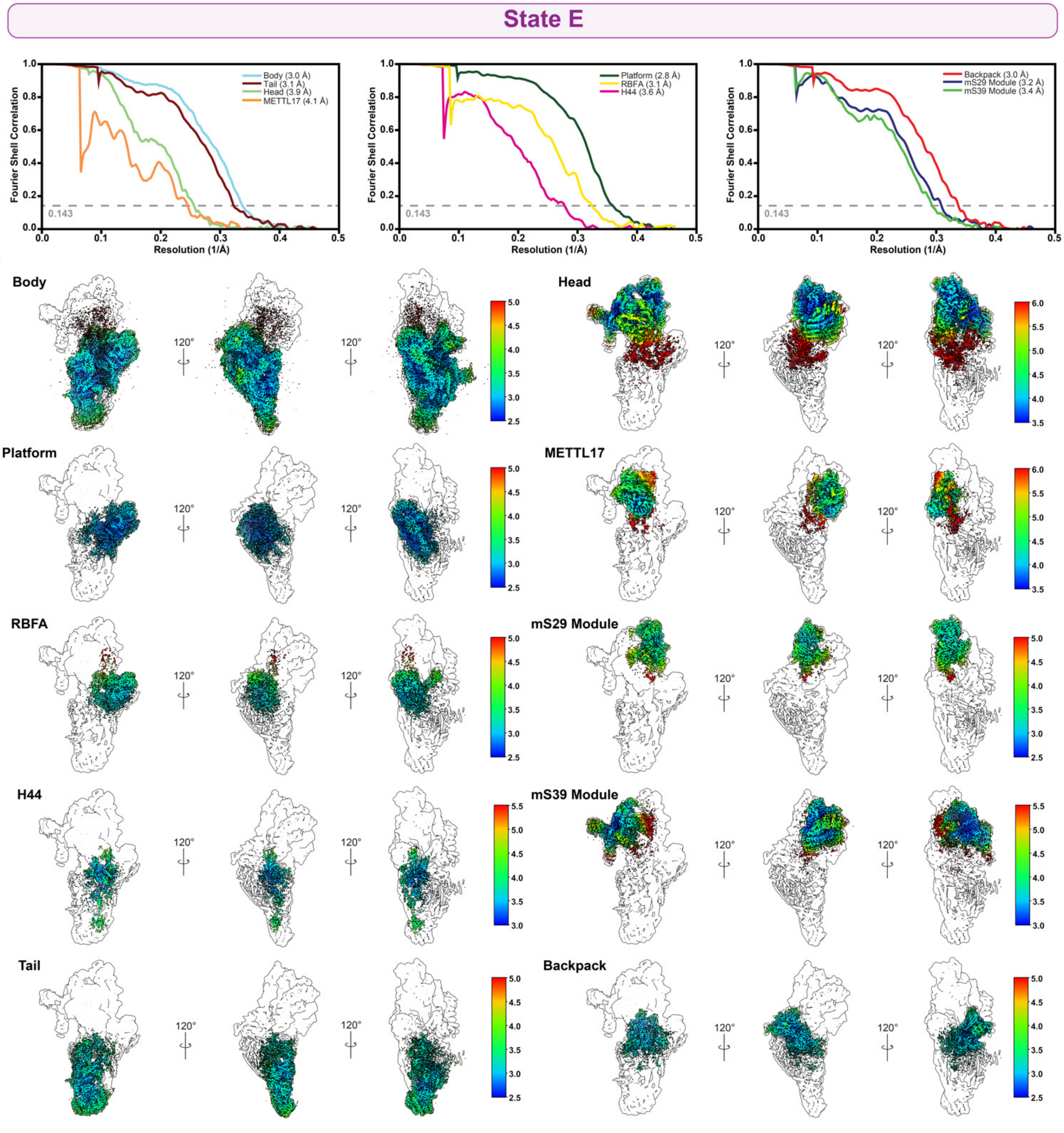

State E
a
Body (3.0 Å)
Tail (3.1 Å)
Head (3.9 Å)
METTL17 (4.1 Å)
Platform (2.8 Å)
RBFA (3.1 Å)
H44 (3.6 Å)
Backpack (3.0 Å)
mS29 Module (3.2 Å)
mS39 Module (3.4 Å)
Fourier Shell Correlation
Resolution (1/Å)
0.143
b
Body
Head
Platform
METTL17
RBFA
mS29 Module
H44
mS39 Module
Tail
Backpack
120°

Supplementary Fig. 14. Local resolution analysis of human State E.

(**a**) Solvent-corrected FSC curves for each focused refinement determined at FSC=0.143 via post-processing in Relion. (**b**) Local resolution estimation (calculated in Relion) for each focused map in context with the rest of the reconstruction.

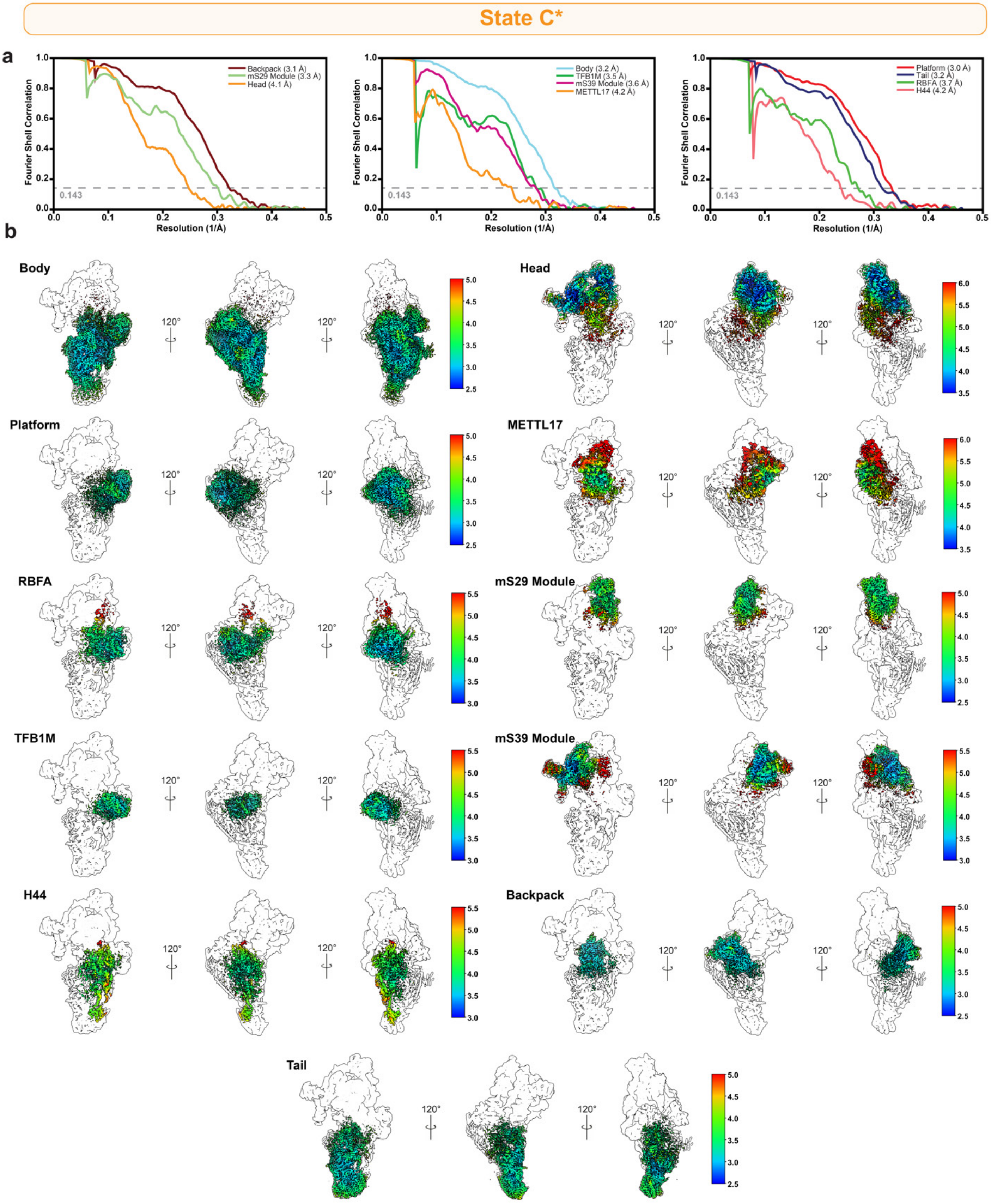
State C*
a
Backpack (3.1 Å)
mS29 Module (3.3 Å)
Head (4.1 Å)
Body (3.2 Å)
TFB1M (3.5 Å)
mS39 Module (3.6 Å)
METTL17 (4.2 Å)
Platform (3.0 Å)
Tail (3.2 Å)
RBFA (3.7 Å)
H44 (4.2 Å)
Fourier Shell Correlation
Resolution (1/Å)
0.143
b
Body
Head
Platform
METTL17
RBFA
mS29 Module
TFB1M
mS39 Module
H44
Backpack
Tail
120°

Supplementary Fig. 15. Local resolution analysis of human State C*.

(**a**) Solvent-corrected FSC curves for each focused refinement determined at FSC=0.143 via post-processing in Relion. (**b**) Local resolution estimation (calculated in Relion) for each focused map in context with the rest of the reconstruction.

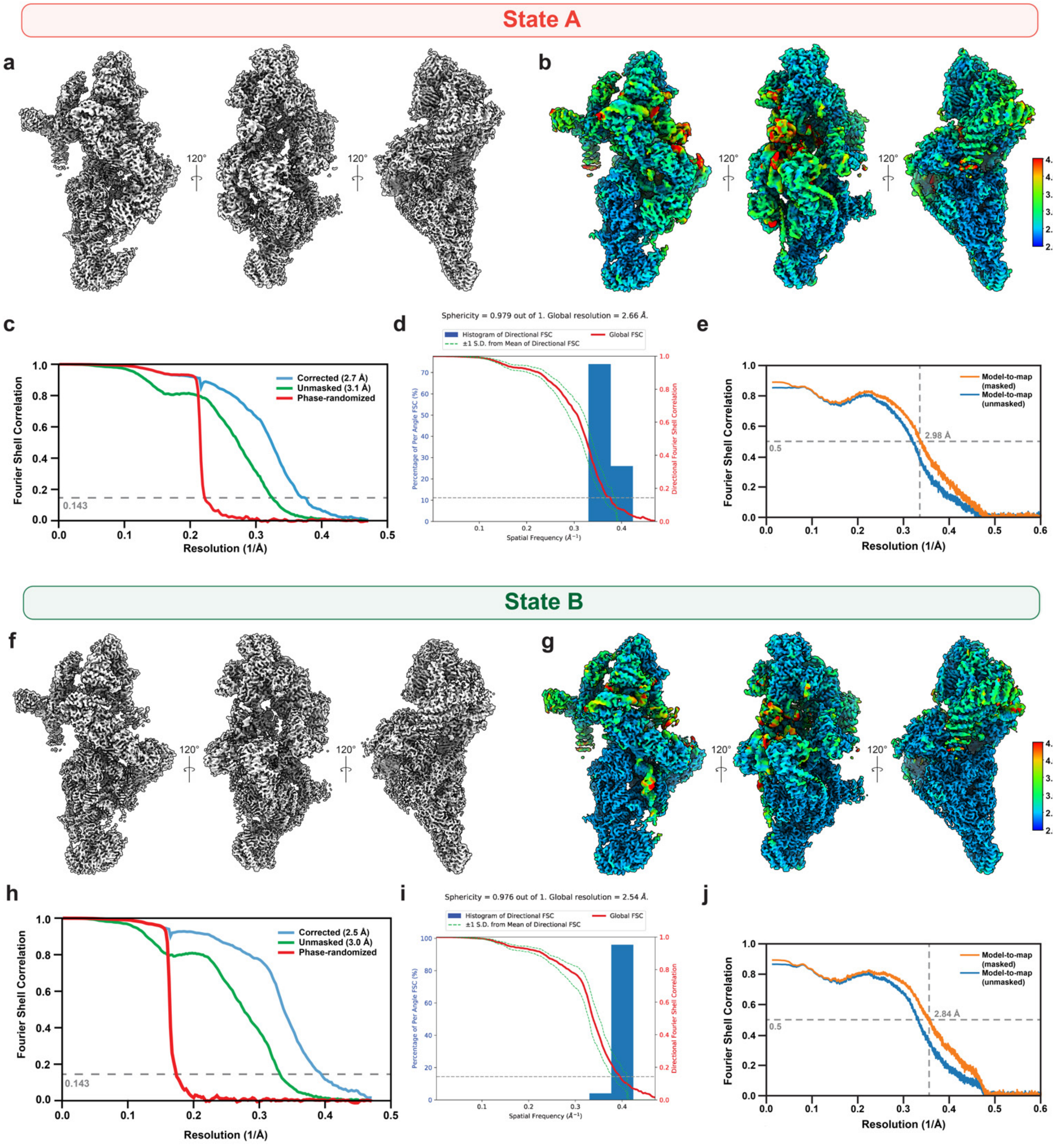
State A
a
120°
120°
b
120°
120°
4.5
4.0
3.5
3.0
2.5
2.0
c
Fourier Shell Correlation
Corrected (2.7 Å)
Unmasked (3.1 Å)
Phase-randomized
0.143
Resolution (1/Å)
d
Sphericity = 0.979 out of 1. Global resolution = 2.66 Å.
Histogram of Directional FSC
±1 S.D. from Mean of Directional FSC
Global FSC
Percentage of Per Angle FSC (%)
Directional Fourier Shell Correlation
Spatial Frequency (Å⁻¹)
e
Fourier Shell Correlation
Model-to-map (masked)
Model-to-map (unmasked)
2.98 Å
0.5
Resolution (1/Å)
State B
f
120°
120°
g
120°
120°
4.5
4.0
3.5
3.0
2.5
2.0
h
Fourier Shell Correlation
Corrected (2.5 Å)
Unmasked (3.0 Å)
Phase-randomized
0.143
Resolution (1/Å)
i
Sphericity = 0.976 out of 1. Global resolution = 2.54 Å.
Histogram of Directional FSC
±1 S.D. from Mean of Directional FSC
Global FSC
Percentage of Per Angle FSC (%)
Directional Fourier Shell Correlation
Spatial Frequency (Å⁻¹)
j
Fourier Shell Correlation
Model-to-map (masked)
Model-to-map (unmasked)
2.84 Å
0.5
Resolution (1/Å)

Supplementary Fig. 16. Validation of composite maps for human States A and B.

(**a**) Different views of the composite map of State A. (**b**) Different views of the composite map of State A filtered and colored according to local resolution as calculated by cryoSPARC. (**c**) FSC curves for the State A composite map as calculated in Relion. Reported resolutions were determined at FSC=0.143. (**d**) 3DFSC for the State A composite map calculated using the Remote 3DFSC Processing Server[1]. (**e**) Map-to-model correlation between the State A composite map and the State A model. (**f**) Different views of the composite map of State B. (**g**) Different views of the composite map of State B filtered and colored according to local resolution as calculated by cryoSPARC. (**h**) FSC curves for the State B composite map as calculated in Relion. Reported resolutions were determined at FSC=0.143. (**i**) 3DFSC for the State B composite map calculated using the Remote 3DFSC Processing Server[1]. (**j**) Map-to-model correlation between the State B composite map and the State B model.

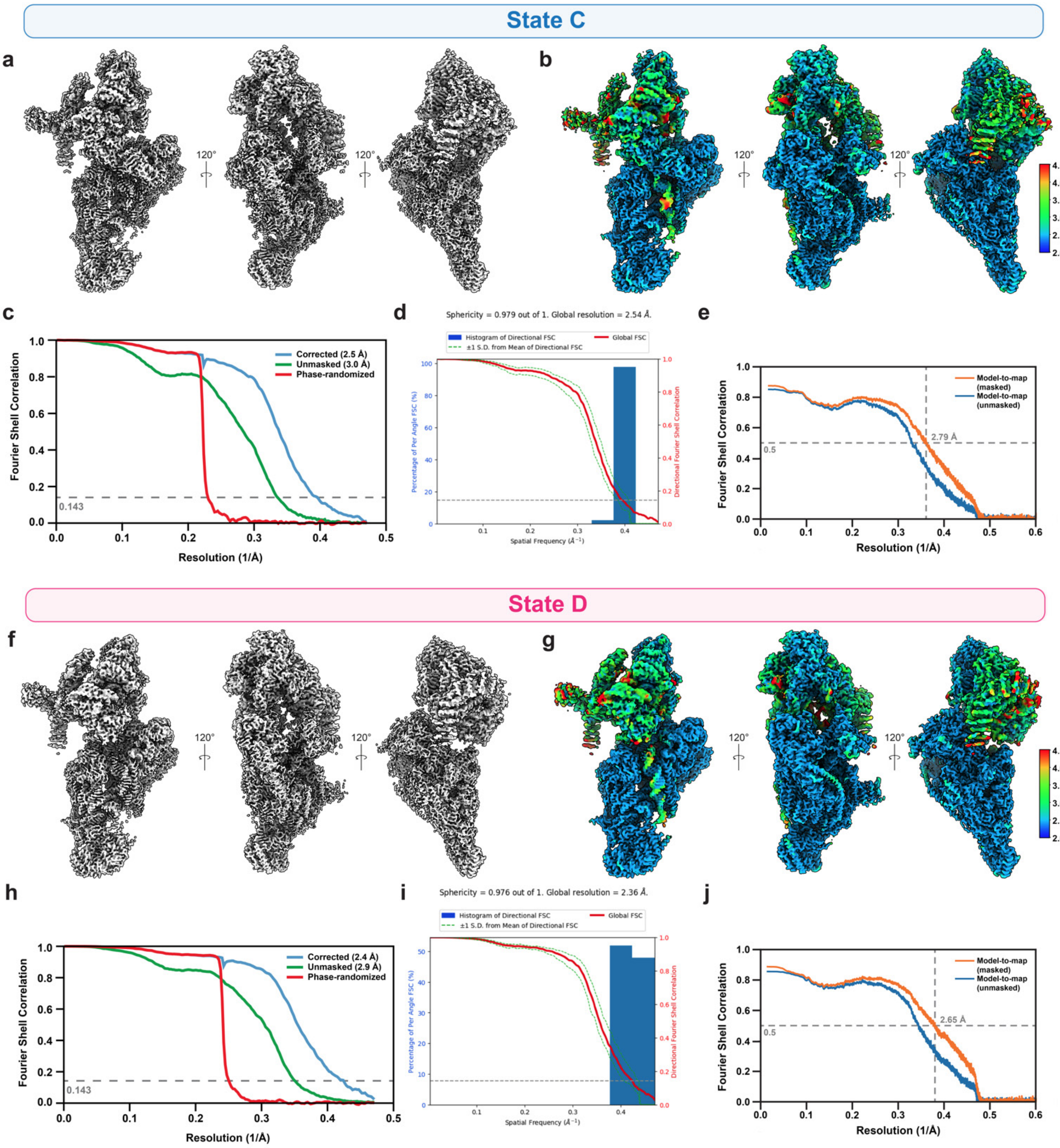

State C
a
120°
120°
b
120°
120°
4.5
4.0
3.5
3.0
2.5
2.0
c
Fourier Shell Correlation
Resolution (1/Å)
Corrected (2.5 Å)
Unmasked (3.0 Å)
Phase-randomized
0.143
d
Sphericity = 0.979 out of 1. Global resolution = 2.54 Å.
Histogram of Directional FSC
±1 S.D. from Mean of Directional FSC
Global FSC
Percentage of Per Angle FSC (%)
Directional Fourier Shell Correlation
Spatial Frequency (Å⁻¹)
e
Model-to-map (masked)
Model-to-map (unmasked)
2.79 Å
0.5
Fourier Shell Correlation
Resolution (1/Å)
State D
f
120°
120°
g
120°
120°
4.5
4.0
3.5
3.0
2.5
2.0
h
Fourier Shell Correlation
Resolution (1/Å)
Corrected (2.4 Å)
Unmasked (2.9 Å)
Phase-randomized
0.143
i
Sphericity = 0.976 out of 1. Global resolution = 2.36 Å.
Histogram of Directional FSC
±1 S.D. from Mean of Directional FSC
Global FSC
Percentage of Per Angle FSC (%)
Directional Fourier Shell Correlation
Spatial Frequency (Å⁻¹)
j
Model-to-map (masked)
Model-to-map (unmasked)
2.65 Å
0.5
Fourier Shell Correlation
Resolution (1/Å)

Supplementary Fig. 17. Validation of composite maps for human States C and D. (**a**) Different views of the composite map of State C. (**b**) Different views of the composite map of State C filtered and colored according to local resolution as calculated by cryoSPARC. (**c**) FSC curves for the State C composite map as calculated in Relion. Reported resolutions were determined at FSC=0.143. (**d**) 3DFSC for the State C composite map calculated using the Remote 3DFSC Processing Server[1]. (**e**) Map-to-model correlation between the State C composite map and the State C model. (**f**) Different views of the composite map of State D. (**g**) Different views of the composite map of State D filtered and colored according to local resolution as calculated by cryoSPARC. (**h**) FSC curves for the State D composite map as calculated in Relion. Reported resolutions were determined at FSC=0.143. (**i**) 3DFSC for the State D composite map calculated using the Remote 3DFSC Processing Server[1]. (**j**) Map-to-model correlation between the State D composite map and the State D model.

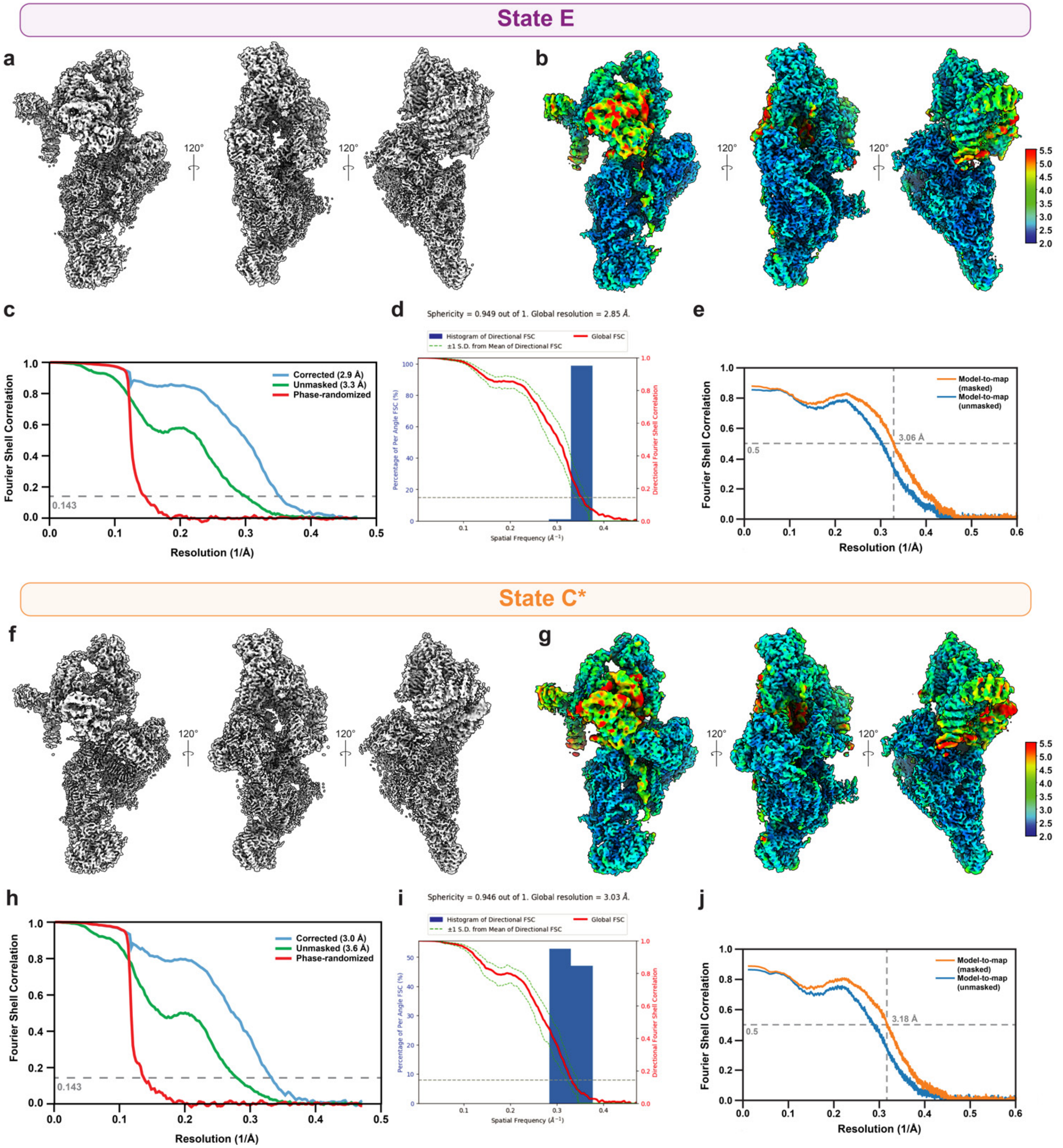
State E
a
120°
120°
b
120°
120°
5.5
5.0
4.5
4.0
3.5
3.0
2.5
2.0
c
Fourier Shell Correlation
Corrected (2.9 Å)
Unmasked (3.3 Å)
Phase-randomized
0.143
Resolution (1/Å)
d
Sphericity = 0.949 out of 1. Global resolution = 2.85 Å.
Histogram of Directional FSC
±1 S.D. from Mean of Directional FSC
Global FSC
Percentage of Per Angle FSC (%)
Directional Fourier Shell Correlation
Spatial Frequency (Å⁻¹)
e
Fourier Shell Correlation
Model-to-map (masked)
Model-to-map (unmasked)
3.06 Å
0.5
Resolution (1/Å)
State C*
f
120°
120°
g
120°
120°
5.5
5.0
4.5
4.0
3.5
3.0
2.5
2.0
h
Fourier Shell Correlation
Corrected (3.0 Å)
Unmasked (3.6 Å)
Phase-randomized
0.143
Resolution (1/Å)
i
Sphericity = 0.946 out of 1. Global resolution = 3.03 Å.
Histogram of Directional FSC
±1 S.D. from Mean of Directional FSC
Global FSC
Percentage of Per Angle FSC (%)
Directional Fourier Shell Correlation
Spatial Frequency (Å⁻¹)
j
Fourier Shell Correlation
Model-to-map (masked)
Model-to-map (unmasked)
3.18 Å
0.5
Resolution (1/Å)

Supplementary Fig. 18. Validation of composite maps for human States E and C*.

(**a**) Different views of the composite map of State E. (**b**) Different views of the composite map of State E filtered and colored according to local resolution as calculated by cryoSPARC. (**c**) FSC curves for the State E composite map as calculated in Relion. Reported resolutions were determined at FSC=0.143. (**d**) 3DFSC for the State E composite map calculated using the Remote 3DFSC Processing Server[1]. (**e**) Map-to-model correlation between the State E composite map and the State E model. (**f**) Different views of the composite map of State C*. (**g**) Different views of the composite map of State C* filtered and colored according to local resolution as calculated by cryoSPARC. (**h**) FSC curves for the State C* composite map as calculated in Relion. Reported resolutions were determined at FSC=0.143. (**i**) 3DFSC for the State C* composite map calculated using the Remote 3DFSC Processing Server[1]. (**j**) Map-to-model correlation between the State C* composite map and the State C* model.

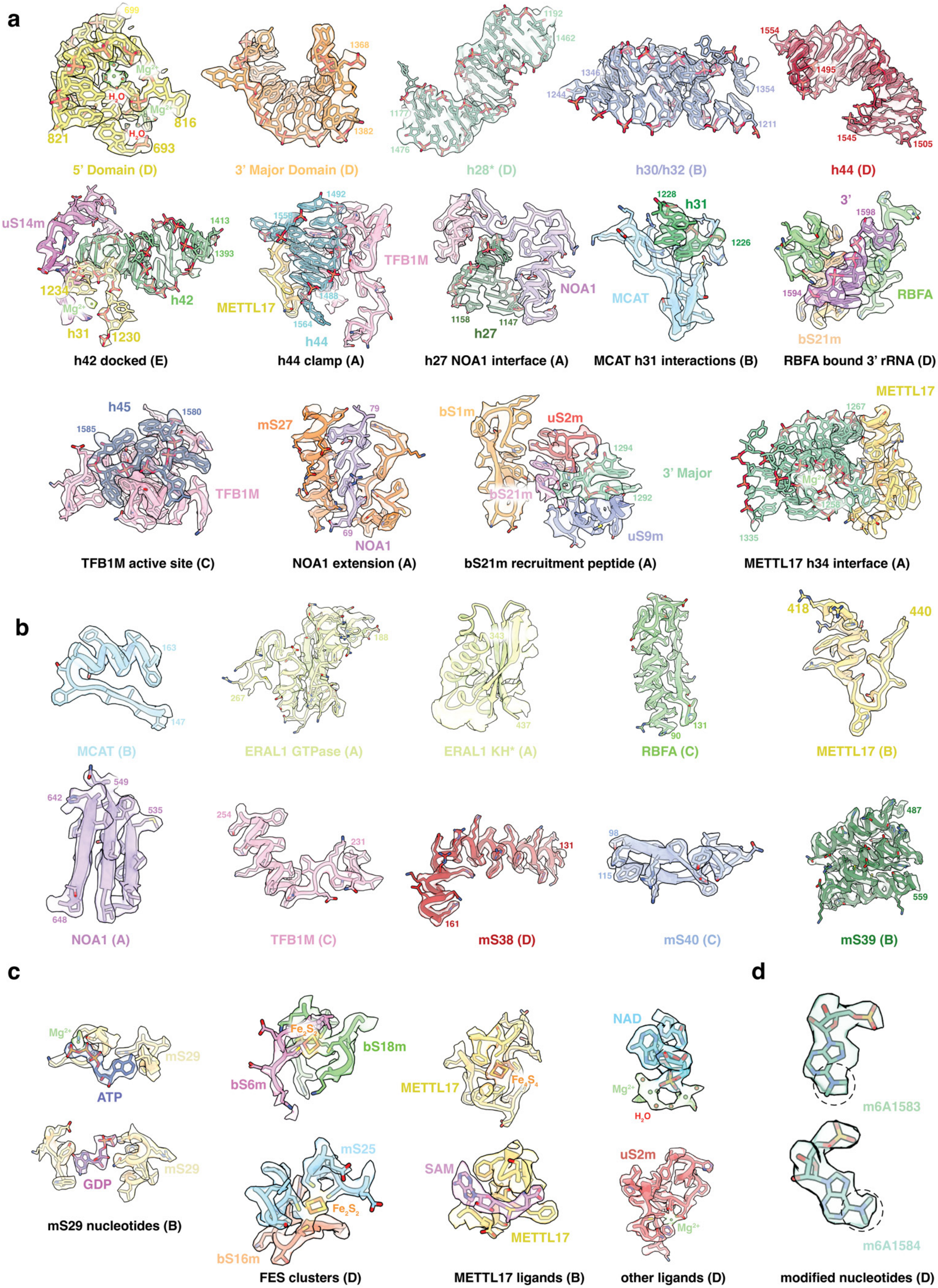
a
5' Domain (D)
3' Major Domain (D)
h28* (D)
h30/h32 (B)
h44 (D)
h42 docked (E)
h44 clamp (A)
h27 NOA1 interface (A)
MCAT h31 interactions (B)
RBFA bound 3' rRNA (D)
TFB1M active site (C)
NOA1 extension (A)
bS21m recruitment peptide (A)
METTL17 h34 interface (A)
b
MCAT (B)
ERAL1 GTPase (A)
ERAL1 KH* (A)
RBFA (C)
METTL17 (B)
NOA1 (A)
TFB1M (C)
mS38 (D)
mS40 (C)
mS39 (B)
c
mS29 nucleotides (B)
FES clusters (D)
METTL17 ligands (B)
other ligands (D)
d
m6A1583
m6A1584
modified nucleotides (D)

Supplementary Fig. 19. Representative cryo-EM densities and models for human structures.

Selections of representative densities of RNA and protein across all states, comprised of (**a**) rRNA and functionally relevant protein-RNA and protein-protein interfaces, (**b**) assembly factors and mitoribosomal proteins, (**c**) ligands, and (**d**) modified nucleotides. The states from which density and models are derived are shown in parentheses below each panel, and * denotes maps that have been filtered to local resolution.

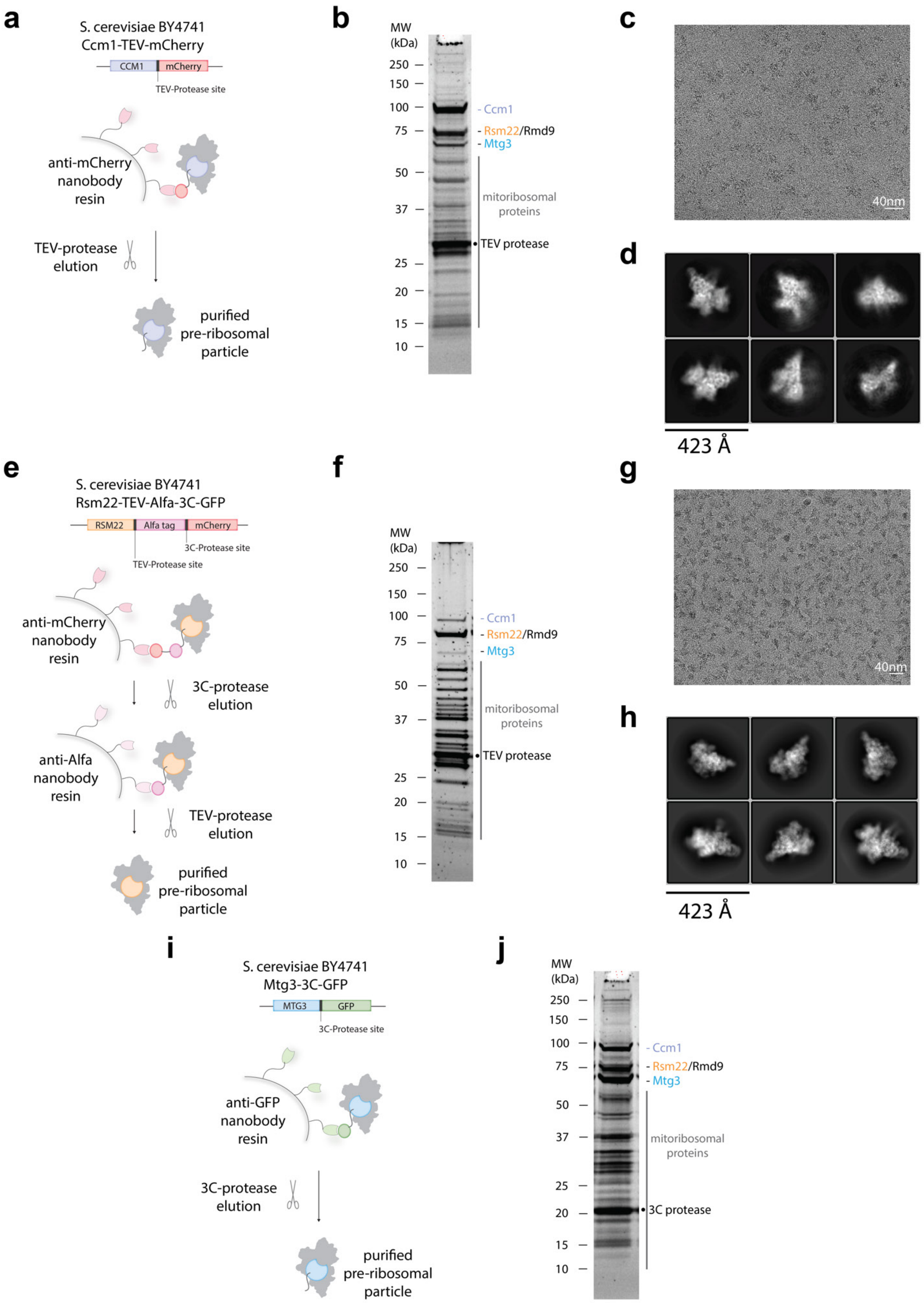
a
S. cerevisiae BY4741
Ccm1-TEV-mCherry
CCM1
mCherry
TEV-Protease site
anti-mCherry nanobody resin
TEV-protease elution
purified pre-ribosomal particle
b
MW (kDa)
250
150
100
75
50
37
25
20
15
10
- Ccm1
- Rsm22/Rmd9
- Mtg3
mitoribosomal proteins
TEV protease
c
40nm
d
423 Å
e
S. cerevisiae BY4741
Rsm22-TEV-Alfa-3C-GFP
RSM22
Alfa tag
mCherry
3C-Protease site
TEV-Protease site
anti-mCherry nanobody resin
3C-protease elution
anti-Alfa nanobody resin
TEV-protease elution
purified pre-ribosomal particle
f
MW (kDa)
250
150
100
75
50
37
25
20
15
10
- Ccm1
- Rsm22/Rmd9
- Mtg3
mitoribosomal proteins
TEV protease
g
40nm
h
423 Å
i
S. cerevisiae BY4741
Mtg3-3C-GFP
MTG3
GFP
3C-Protease site
anti-GFP nanobody resin
3C-protease elution
purified pre-ribosomal particle
j
MW (kDa)
250
150
100
75
50
37
25
20
15
10
- Ccm1
- Rsm22/Rmd9
- Mtg3
mitoribosomal proteins
3C protease

Supplementary Fig. 20. Purification of yeast mtSSU assembly intermediates.

(**a**) Purification scheme for Ccm1-containing mitoribosomal intermediates. (**b**) Representative SDS-PAGE gel of eluted Ccm1 sample. Proteins were identified with LC-MS/MS (Supplementary Data S2). Immunoprecipitation experiments were repeated at least 3 times with similar results. (**c**) Motion corrected cryo-EM micrograph of Ccm1-bound particles representative of 31,995 total micrographs. (**d**) 6 representative 2D class averages for the Ccm1 dataset (4x binned, 4.32 Å/px). (**e**) Purification scheme for Rsm22-containing mitoribosomal intermediates. (**f**) Representative SDS-PAGE gel of eluted Rsm22 sample. Proteins were identified with LC-MS/MS (Supplementary Data S3). Immunoprecipitation experiments were repeated at least 3 times with similar results. (**g**) Motion corrected cryo-EM micrograph of Rsm22-bound particles representative of 14,111 total micrographs. (**h**) 6 representative 2D class averages for the Rsm22 dataset (8x binned, 8.64 Å/px). (**i**) Purification scheme for Mtg3-containing mitoribosomal intermediates. (**j**) Representative SDS-PAGE gel of eluted Mtg3 sample. Proteins were identified with LC-MS/MS (Supplementary Data S4). Immunoprecipitation experiments were repeated at least 3 times with similar results. For gel source data, see Supplementary Fig. 1.

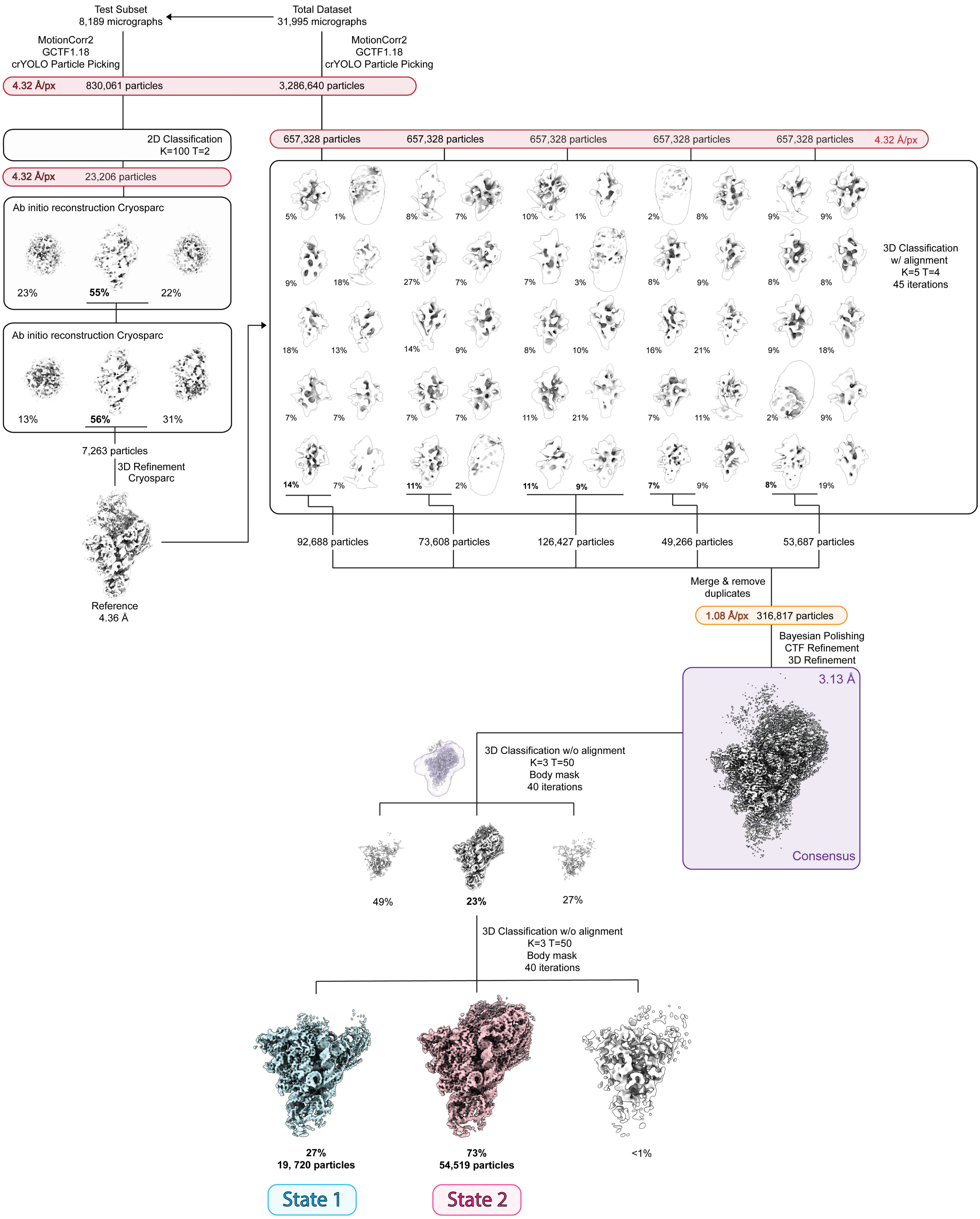

Test Subset
8,189 micrographs
Total Dataset
31,995 micrographs
MotionCorr2
GCTF1.18
crYOLO Particle Picking
MotionCorr2
GCTF1.18
crYOLO Particle Picking
4.32 Å/px
830,061 particles
3,286,640 particles
2D Classification
K=100 T=2
4.32 Å/px
23,206 particles
Ab initio reconstruction Cryosparc
23%
55%
22%
Ab initio reconstruction Cryosparc
13%
56%
31%
7,263 particles
3D Refinement
Cryosparc
Reference
4.36 Å
657,328 particles
657,328 particles
657,328 particles
657,328 particles
657,328 particles
4.32 Å/px
5%
1%
8%
7%
10%
1%
2%
8%
9%
9%
9%
18%
27%
7%
7%
3%
8%
9%
8%
8%
18%
13%
14%
9%
8%
10%
16%
21%
9%
18%
7%
7%
7%
7%
11%
21%
7%
11%
2%
9%
14%
7%
11%
2%
11%
9%
7%
9%
8%
19%
3D Classification
w/ alignment
K=5 T=4
45 iterations
92,688 particles
73,608 particles
126,427 particles
49,266 particles
53,687 particles
Merge & remove
duplicates
1.08 Å/px
316,817 particles
Bayesian Polishing
CTF Refinement
3D Refinement
3.13 Å
Consensus
3D Classification w/o alignment
K=3 T=50
Body mask
40 iterations
49%
23%
27%
3D Classification w/o alignment
K=3 T=50
Body mask
40 iterations
27%
19, 720 particles
73%
54,519 particles
<1%
State 1
State 2

Supplementary Fig. 21. Initial cryo-EM processing of yeast Ccm1-containing particles.

Workflow for data processing and identification of two assembly states, States 1 and 2. Reported resolutions (FSC=0.143) from Relion post-processing are reported next to individual maps.

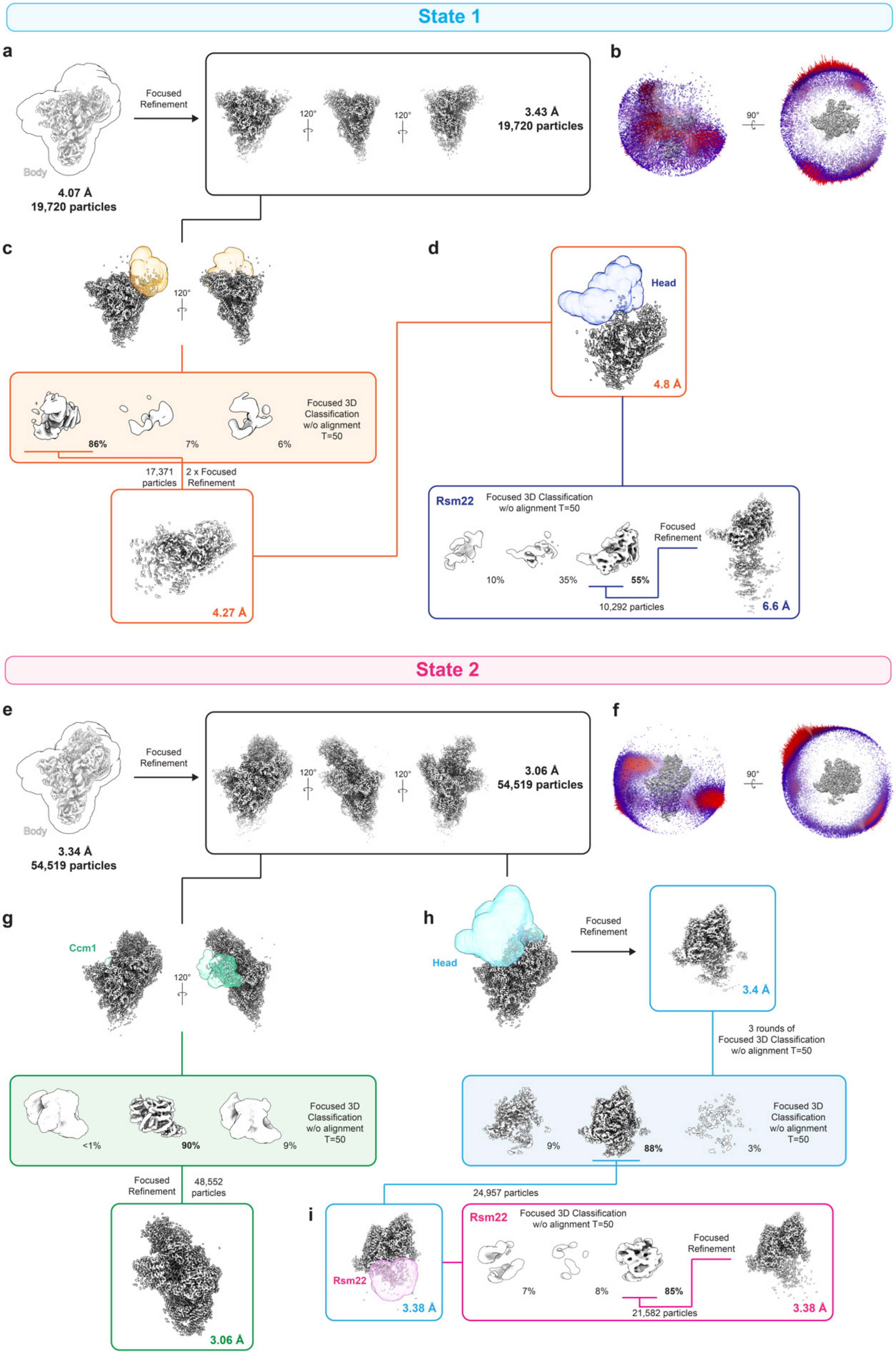

State 1
a
Body
4.07 Å
19,720 particles
Focused Refinement
120°
120°
3.43 Å
19,720 particles
b
90°
c
120°
Focused 3D Classification w/o alignment T=50
86%
7%
6%
17,371 particles
2 x Focused Refinement
4.27 Å
d
Head
4.8 Å
Rsm22
Focused 3D Classification w/o alignment T=50
Focused Refinement
10%
35%
55%
10,292 particles
6.6 Å
State 2
e
Body
3.34 Å
54,519 particles
Focused Refinement
120°
120°
3.06 Å
54,519 particles
f
90°
g
Ccm1
120°
Focused 3D Classification w/o alignment T=50
<1%
90%
9%
Focused Refinement
48,552 particles
3.06 Å
h
Head
Focused Refinement
3.4 Å
3 rounds of Focused 3D Classification w/o alignment T=50
9%
88%
3%
Focused 3D Classification w/o alignment T=50
24,957 particles
i
Rsm22
3.38 Å
Rsm22
Focused 3D Classification w/o alignment T=50
Focused Refinement
7%
8%
85%
21,582 particles
3.38 Å

Supplementary Fig. 22. Cryo-EM processing of yeast States 1 and 2.

(**a**) Particles identified as State 1 were refined using a mask on the body domain. (**b**) Euler angle distribution of the overall (body) refinement for State 1. (**c**) Particles were classified using a mask on the platform and subsequently refined. (**d**) Classification of these particles on the head domain and Rsm22. (**e**) Particles identified as State 2 were refined using a mask on the body domain. (**f**) Euler angle distribution of the overall (body) refinement for State 2. (**g**) Particles were classified using a mask on Ccm1 and subsequently refined using a larger mask encompassing most of the body. (**h**) Particles were refined with a mask on the head domain and further classified. (**i**) To improve fragmented density corresponding to Rsm22, this region was subjected to an additional round of masked classification and refinement. Reported resolutions (FSC=0.143) from Relion post-processing are reported next to individual focused maps. Masks used are color coded.

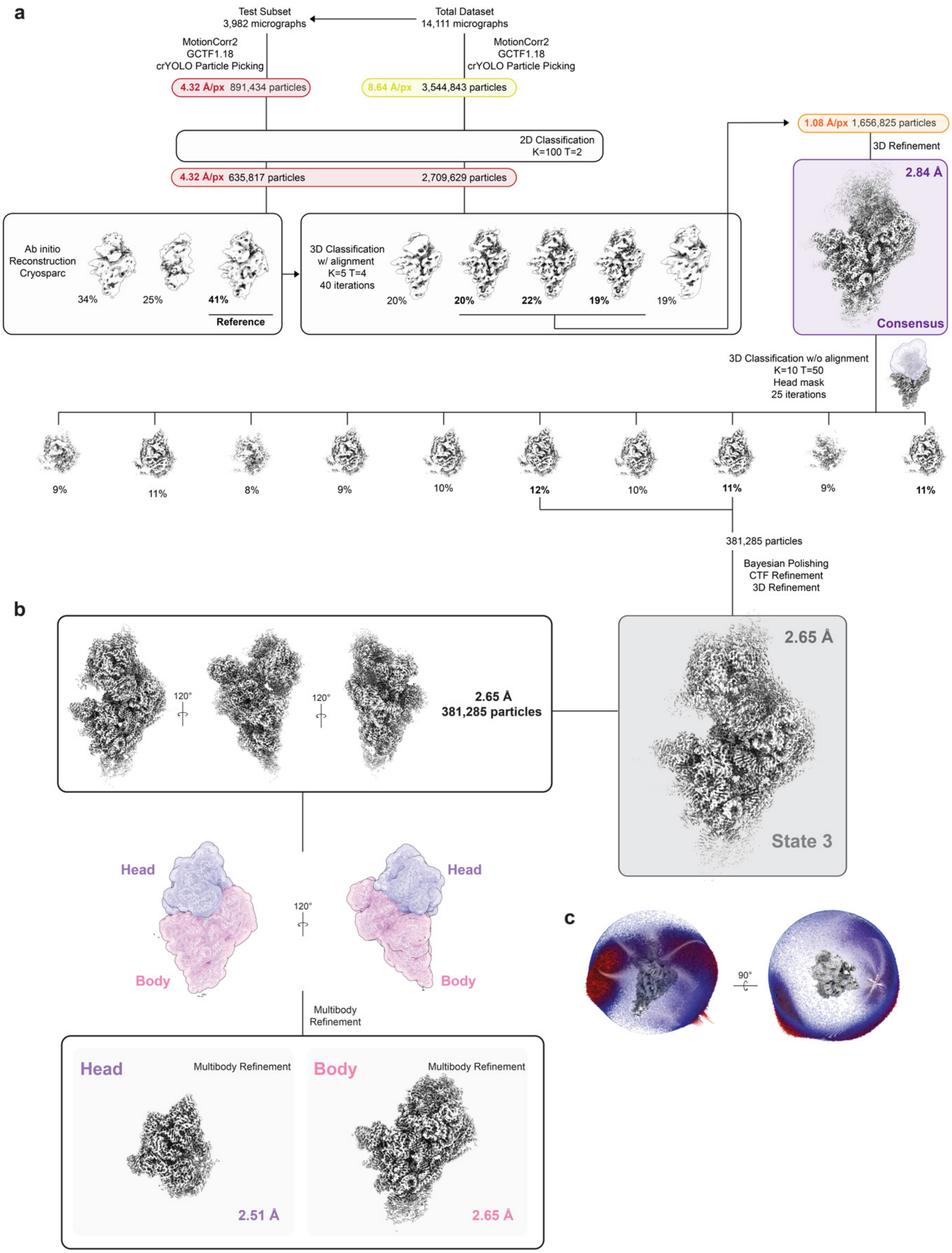

a
Test Subset
3,982 micrographs
Total Dataset
14,111 micrographs
MotionCorr2
GCTF1.18
crYOLO Particle Picking
MotionCorr2
GCTF1.18
crYOLO Particle Picking
4.32 Å/px 891,434 particles
8.64 Å/px 3,544,843 particles
1.08 Å/px 1,656,825 particles
3D Refinement
2D Classification
K=100 T=2
4.32 Å/px 635,817 particles
2,709,629 particles
2.84 Å
Ab initio
Reconstruction
Cryosparc
34%
25%
41%
Reference
3D Classification
w/ alignment
K=5 T=4
40 iterations
20%
20%
22%
19%
19%
Consensus
3D Classification w/o alignment
K=10 T=50
Head mask
25 iterations
9%
11%
8%
9%
10%
12%
10%
11%
9%
11%
381,285 particles
Bayesian Polishing
CTF Refinement
3D Refinement
b
120°
120°
2.65 Å
381,285 particles
2.65 Å
State 3
Head
Head
120°
Body
Body
Multibody
Refinement
Head
Multibody Refinement
2.51 Å
Body
Multibody Refinement
2.65 Å
c
90°

Supplementary Fig. 23. Cryo-EM processing of yeast Rsm22-containing particles (State 3).

(**a**) Workflow for initial data processing to identify particles corresponding to State 3, resulting in a 2.65 Å consensus map. (**b**) Three views of the consensus map. The resolution of the head domain was improved using multibody refinement. (**c**) Euler angle distribution of the overall (body) refinement for State 3. Reported resolutions (FSC=0.143) from Relion post-processing are reported next to individual focused maps. Masks used are color coded.

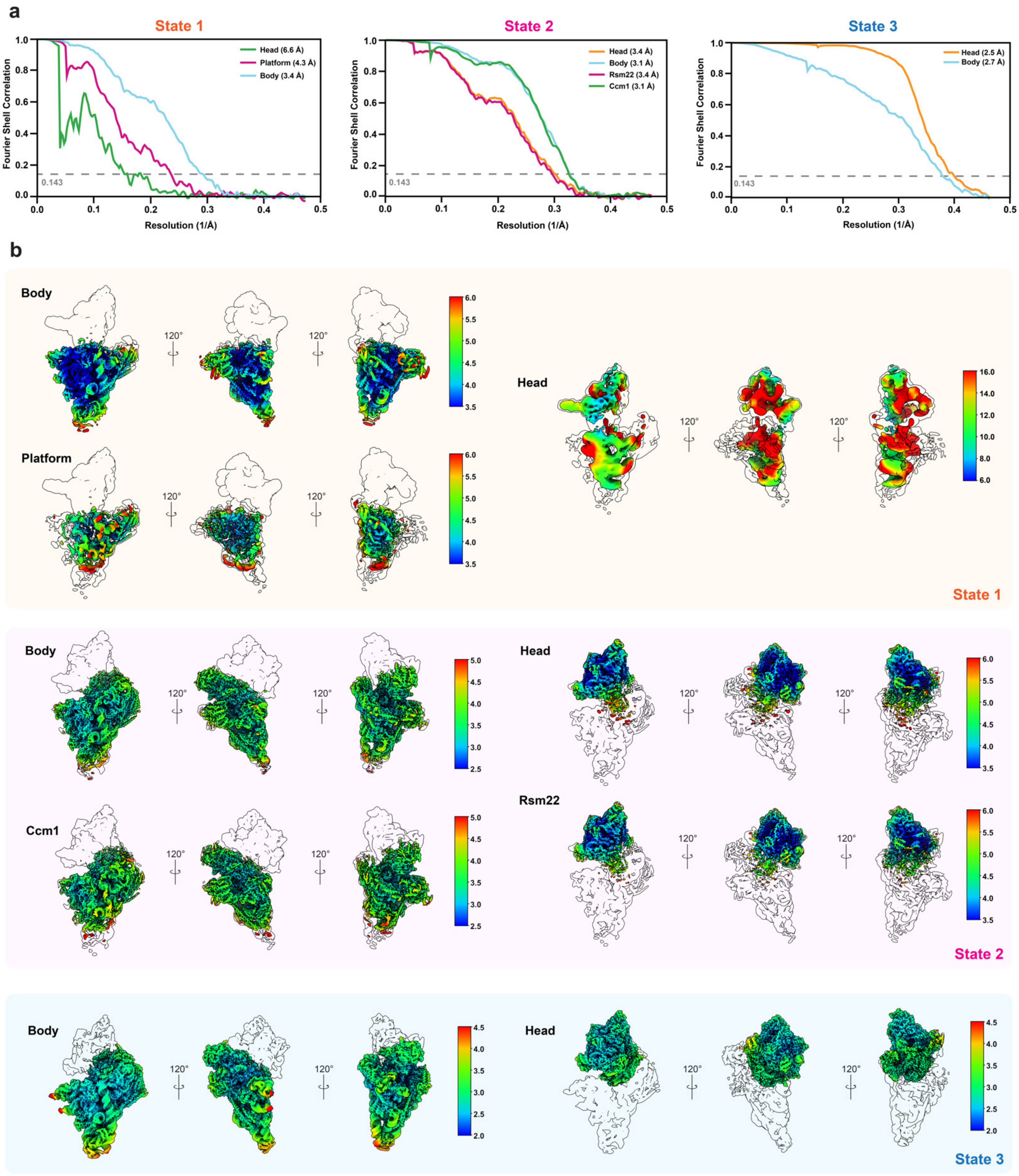
a
State 1
Head (6.6 Å)
Platform (4.3 Å)
Body (3.4 Å)
Fourier Shell Correlation
Resolution (1/Å)
0.143
State 2
Head (3.4 Å)
Body (3.1 Å)
Rsm22 (3.4 Å)
Ccm1 (3.1 Å)
State 3
Head (2.5 Å)
Body (2.7 Å)
b
Body
Platform
Head
120°
State 1
Body
Head
Ccm1
Rsm22
State 2
Body
Head
State 3

Supplementary Fig. 24. Local resolution analysis of yeast reconstructions.

(**a**) Solvent-corrected FSC curves for each focused refinement determined at FSC=0.143 via post-processing in Relion. (**b**) Local resolution estimation (calculated in Relion) for each focused map in context with the rest of the reconstruction.

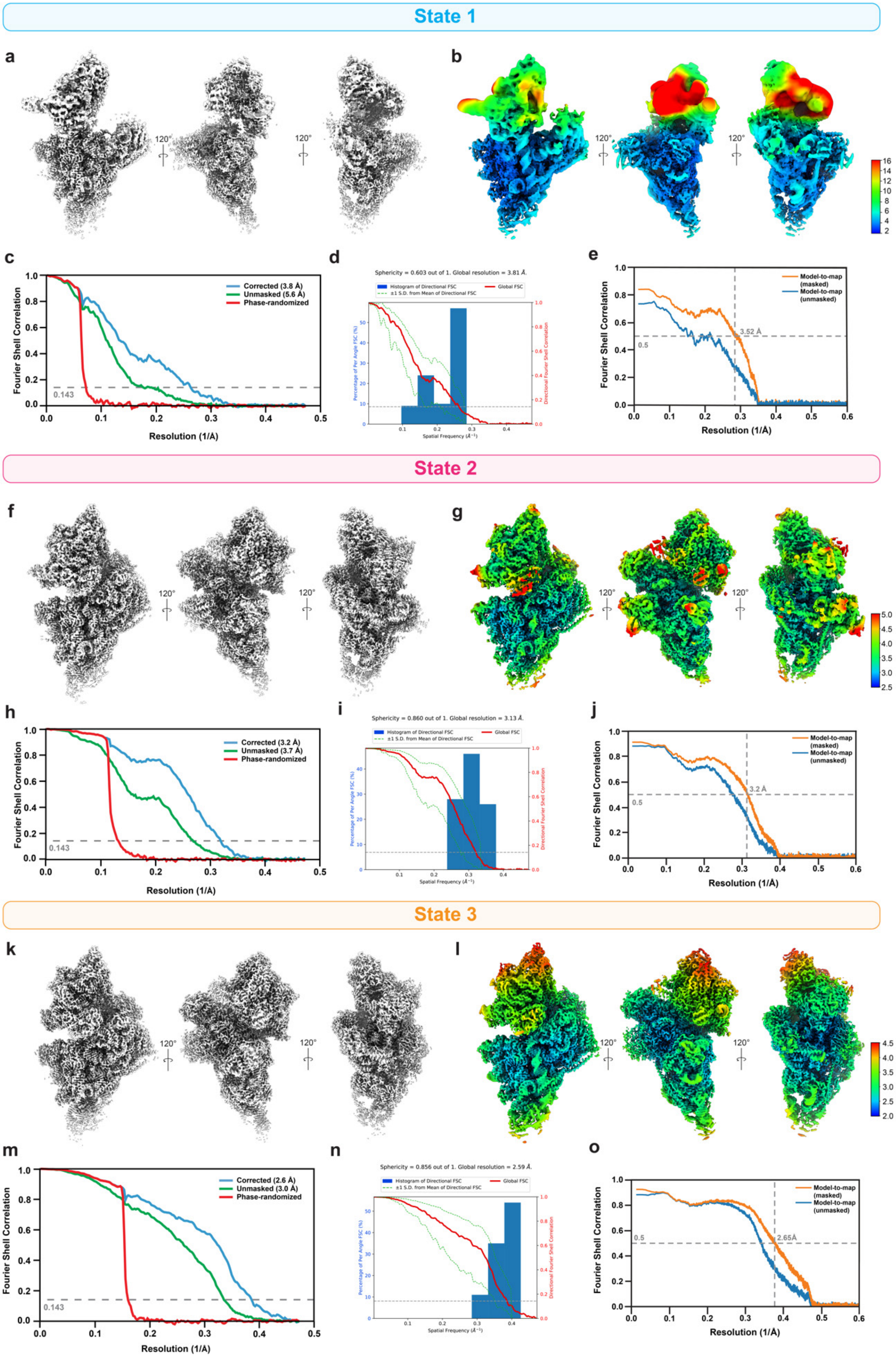
State 1
a
b
120°
120°
c
Fourier Shell Correlation
Resolution (1/Å)
Corrected (3.8 Å)
Unmasked (5.6 Å)
Phase-randomized
0.143
d
Sphericity = 0.603 out of 1. Global resolution = 3.81 Å.
Histogram of Directional FSC
±1 S.D. from Mean of Directional FSC
Global FSC
Percentage of Per Angle FSC (%)
Directional Fourier Shell Correlation
Spatial Frequency (Å⁻¹)
e
Model-to-map (masked)
Model-to-map (unmasked)
3.52 Å
0.5
State 2
f
g
h
Corrected (3.2 Å)
Unmasked (3.7 Å)
Phase-randomized
i
Sphericity = 0.860 out of 1. Global resolution = 3.13 Å.
j
3.2 Å
State 3
k
l
m
Corrected (2.6 Å)
Unmasked (3.0 Å)
Phase-randomized
n
Sphericity = 0.856 out of 1. Global resolution = 2.59 Å.
o
2.65Å

Supplementary Fig. 25. Validation of composite maps for yeast States 1-3.

(**a**) Different views of the composite map of State 1. (**b**) Different views of the composite map of State 1 filtered and colored according to local resolution as calculated by cryoSPARC. (**c**) FSC curves for the State 1 composite map as calculated in Relion. Reported resolutions were determined at FSC=0.143. (**d**) 3DFSC for the State 1 composite map calculated using the Remote 3DFSC Processing Server[1]. (**e**) Map-to-model correlation between the State 1 composite map and the State 1 model. (**f**) Different views of the composite map of State 2. (**g**) Different views of the composite map of State 2 filtered and colored according to local resolution as calculated by cryoSPARC. (**h**) FSC curves for the State 2 composite map as calculated in Relion. Reported resolutions were determined at FSC=0.143. (**i**) 3DFSC for the State 2 composite map calculated using the Remote 3DFSC Processing Server[1]. (**j**) Map-to-model correlation between the State 2 composite map and the State 2 model. (**k**) Different views of the composite map of State 3. (**l**) Different views of the composite map of State 3 filtered and colored according to local resolution as calculated by cryoSPARC. (**m**) FSC curves for the State 3 composite map as calculated in Relion. Reported resolutions were determined at FSC=0.143. (**n**) 3DFSC for the State 3 composite map calculated using the Remote 3DFSC Processing Server[1]. (**o**) Map-to-model correlation between the State 3 composite map and the State 3 model.

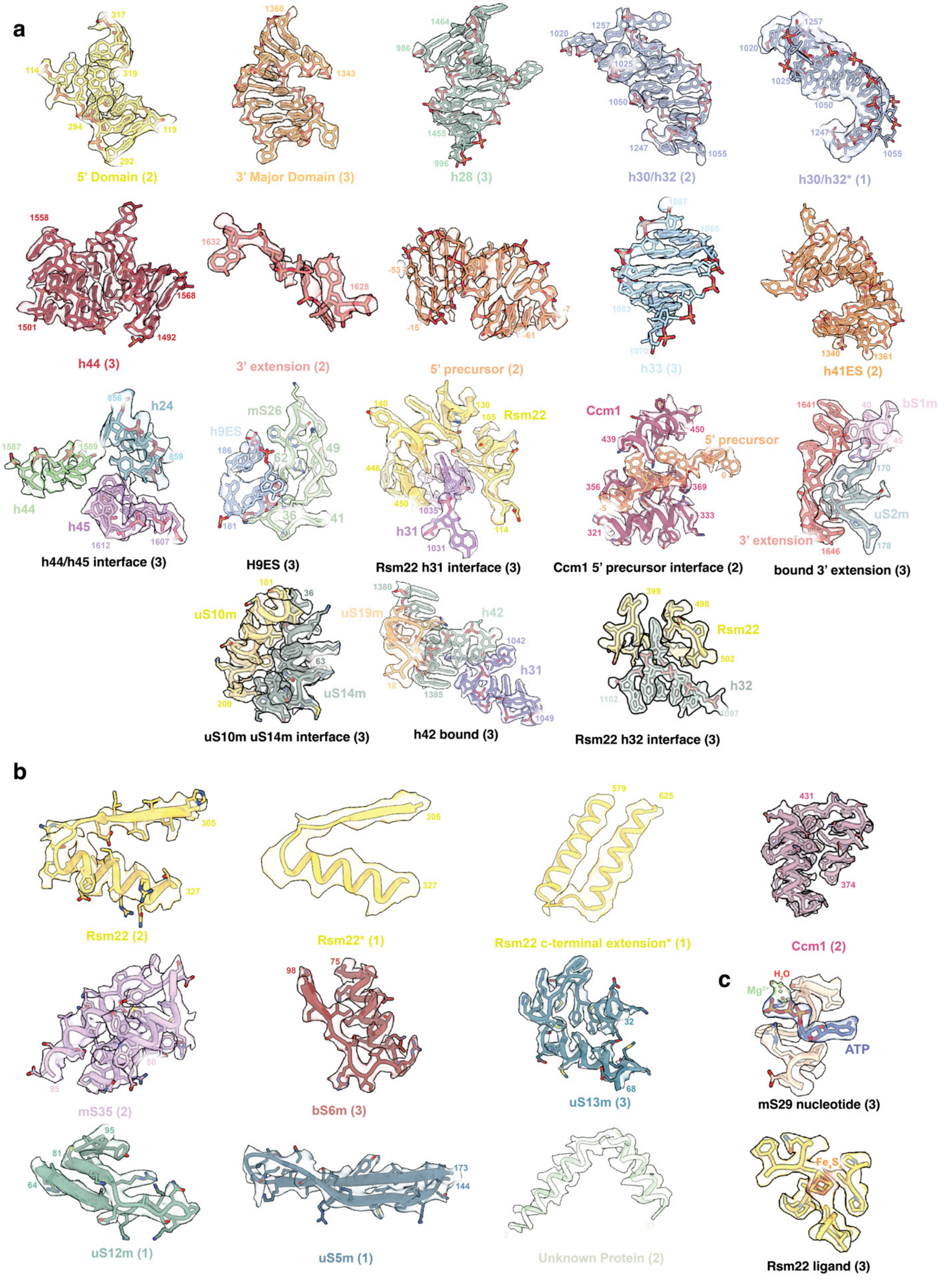
a
5' Domain (2)
3' Major Domain (3)
h28 (3)
h30/h32 (2)
h30/h32* (1)
h44 (3)
3' extension (2)
5' precursor (2)
h33 (3)
h41ES (2)
h44/h45 interface (3)
H9ES (3)
Rsm22 h31 interface (3)
Ccm1 5' precursor interface (2)
bound 3' extension (3)
uS10m uS14m interface (3)
h42 bound (3)
Rsm22 h32 interface (3)
b
Rsm22 (2)
Rsm22* (1)
Rsm22 c-terminal extension* (1)
Ccm1 (2)
mS35 (2)
bS6m (3)
uS13m (3)
uS12m (1)
uS5m (1)
Unknown Protein (2)
c
mS29 nucleotide (3)
ATP
Rsm22 ligand (3)

Supplementary Fig. 26. Representative cryo-EM densities and models for yeast structures.

Selections of representative densities of RNA and protein across all states, comprised of (**a**) rRNA and functionally relevant protein-RNA and protein-protein interfaces, (**b**) assembly factors and mitoribosomal proteins, and (**c**) ligands. The states from which density and models are derived are shown in parenthesis below each panel, and * denotes maps that have been filtered to local resolution.

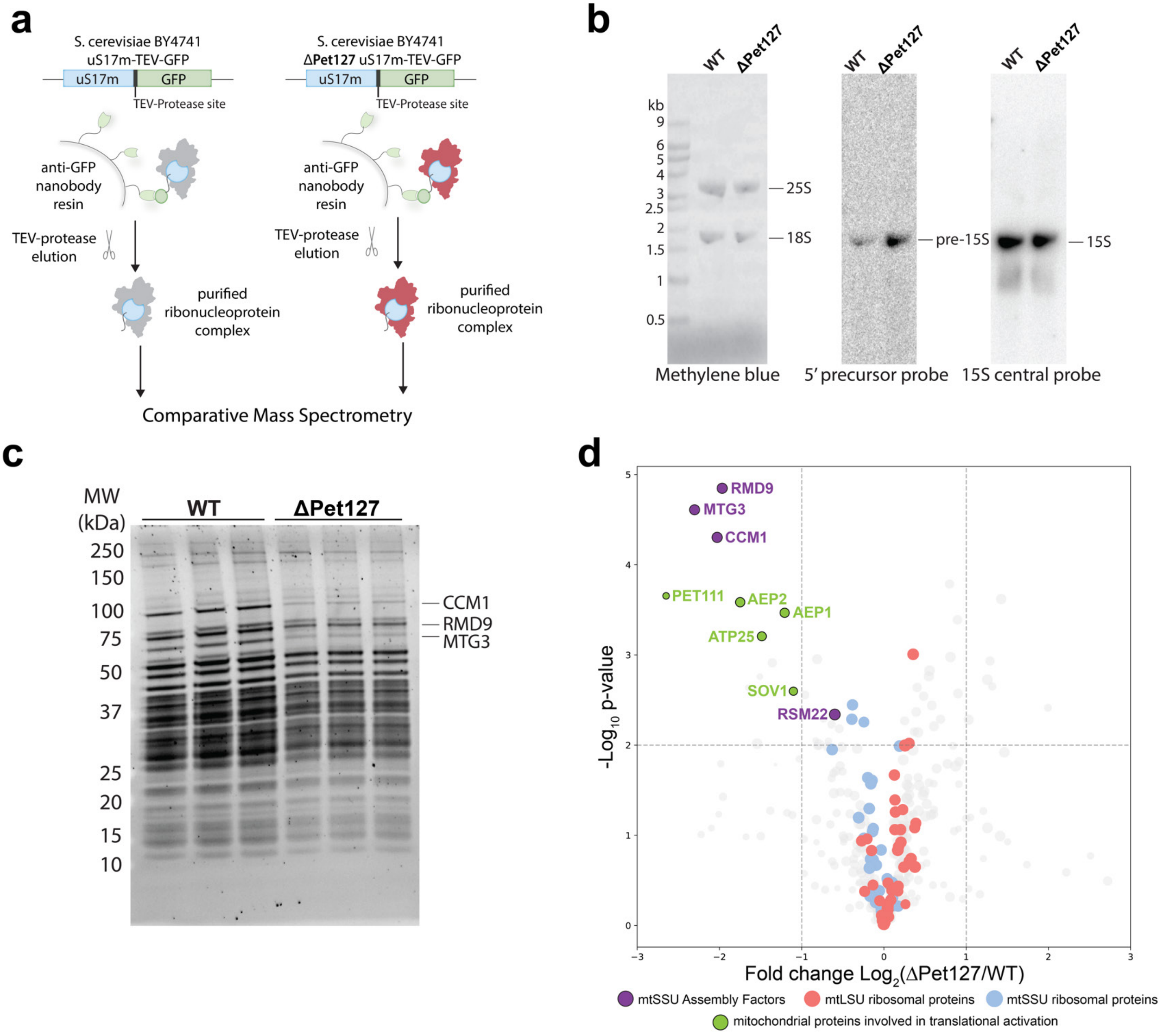
a
S. cerevisiae BY4741
uS17m-TEV-GFP
uS17m
GFP
TEV-Protease site
anti-GFP
nanobody
resin
TEV-protease
elution
purified
ribonucleoprotein
complex
S. cerevisiae BY4741
ΔPet127 uS17m-TEV-GFP
Comparative Mass Spectrometry
b
WT
ΔPet127
kb
9
6
5
4
3
2.5
2
1.5
1
0.5
25S
18S
pre-15S
15S
Methylene blue
5′ precursor probe
15S central probe
c
MW
(kDa)
250
150
100
75
50
37
25
20
15
10
WT
ΔPet127
CCM1
RMD9
MTG3
d
RMD9
MTG3
CCM1
PET111
AEP2
AEP1
ATP25
SOV1
RSM22
-Log10 p-value
Fold change Log2(ΔPet127/WT)
mtSSU Assembly Factors
mtLSU ribosomal proteins
mtSSU ribosomal proteins
mitochondrial proteins involved in translational activation

Supplementary Fig. 27 - Pet127 knockout results in accumulation of pre-15S rRNA and a decrease in bound assembly factors.

(**a**) Experimental scheme for comparative IP-MS/MS in WT and PET127 KO yeast cells. (**b**) Northern blots of extracted RNA after immunoprecipitation of uS17m-bound complexes. Northern blotting experiments were performed twice with similar results. (**c**) SDS-PAGE gel of eluted complexes after uS17m pulldown in triplicate. Assembly factors identified by mass spectrometry that are significantly decreased in Pet127 KO samples are labeled. (**d**) Volcano plot for comparative IP-MS/MS of WT and PET127 KO cells using uS17m as bait. IP-MS/MS experiments were performed in triplicate for each condition (n=3 biologically independent experiments). A 2-sided student t-test was used to determine p-values for changes in protein abundance between WT and PET127 KO mtSSU purification experiments. Individual proteins are shown as circles, with blue circles indicating mtSSU proteins, orange circles indicating mtLSU proteins, green circles indicating proteins involved in translational activation, and purple circles indicating mtSSU assembly factors. Raw data is available in Supplementary Data S5. For gel source data, see Supplementary Fig. 1.

SUPPLEMENTARY REFERENCES

1. Tan, Y. Z. *et al.* Addressing preferred specimen orientation in single-particle cryo-EM through tilting. *Nat. Methods* **14,** 793–796 (2017).
