# Supplementary material for: Principles of mitoribosomal small subunit assembly in eukaryotes
Source: Nature. 2022 Dec 8;614(7946):175–81. doi: 10.1038/s41586-022-05621-0 (PMC9892005; doi:10.1038/s41586-022-05621-0)
Supplement: Supplementary file 2 — Reporting Summary [file 41586_2022_5621_MOESM2_ESM.pdf]

nature portfolio

Corresponding author(s): Sebastian Klinge

Last updated by author(s): Nov 21, 2022

Reporting Summary

Nature Portfolio wishes to improve the reproducibility of the work that we publish. This form provides structure for consistency and transparency in reporting. For further information on Nature Portfolio policies, see our Editorial Policies and the Editorial Policy Checklist.

Statistics

For all statistical analyses, confirm that the following items are present in the figure legend, table legend, main text, or Methods section.

n/a	Confirmed	
☐	☒	The exact sample size (n) for each experimental group/condition, given as a discrete number and unit of measurement
☐	☒	A statement on whether measurements were taken from distinct samples or whether the same sample was measured repeatedly
☐	☒	The statistical test(s) used AND whether they are one- or two-sided<br>*Only common tests should be described solely by name; describe more complex techniques in the Methods section.*
☒	☐	A description of all covariates tested
☐	☒	A description of any assumptions or corrections, such as tests of normality and adjustment for multiple comparisons
☐	☒	A full description of the statistical parameters including central tendency (e.g. means) or other basic estimates (e.g. regression coefficient) AND variation (e.g. standard deviation) or associated estimates of uncertainty (e.g. confidence intervals)
☐	☒	For null hypothesis testing, the test statistic (e.g. F, t, r) with confidence intervals, effect sizes, degrees of freedom and P value noted<br>*Give P values as exact values whenever suitable.*
☒	☐	For Bayesian analysis, information on the choice of priors and Markov chain Monte Carlo settings
☒	☐	For hierarchical and complex designs, identification of the appropriate level for tests and full reporting of outcomes
☒	☐	Estimates of effect sizes (e.g. Cohen's d, Pearson's r), indicating how they were calculated

Our web collection on statistics for biologists contains articles on many of the points above.

Software and code

Policy information about availability of computer code

Data collection	Cryo-EM datasets were collected using SerialEM (v. 3.8) on an FEI Titan Krios (FEI/Thermofisher) microscope using an energy filter at a slit width of 20 eV operating at 300 kV and 64,000x magnification. 3 datasets were collected at super-resolution pixel size of 0.54 Å/px, with defocus values ranging from -0.7 to -2.5 µm. Electron dose ranged between 51 and 62 electrons/Å2 over 40-42 frames. For comparative IP-MS/MS experiments, samples were analyzed by reversed phase nano-LC-MS/MS using a Fusion Lumos (Thermo Scientific).
Data analysis	Cryo-EM movies were aligned and averaged in Relion (v. 3.1.0) using the MotionCor2-like algorithm implemented in Relion , and Contrast transfer function parameters were estimated using GCTF1.18. Particles were picked using crYOLO (v. 1.7.6) and Relion was used for subsequent classification and refinement steps. Models were manually built in Coot (v. 0.9) using starting models from the PDB (6RW4, 2C2N,6AAX,5MRC) and from the AlphaFold database. Refinement was performed using PHENIX 1.19. Final models were validated using MolProbity, EMRinger, and phenix.rna_validate in PHENIX 1.19. For comparative IP-MS/MS experiments, Data were quantified and searched against the S. cerevisiae or H. sapiens Uniprot protein database (2019) concatenated with the MS2-protein sequence and common contaminations. For the search and quantitation, MaxQuant v. 2.0.3.0 was used. Oxidation of methionine and protein N-terminal acetylation were allowed as variable modifications and all cysteines were treated as being carbamidomethylated. The 'match between runs' option was enabled, and false discovery rates for proteins and peptides were set to 1% and 2% respectively. Protein abundances were expressed as LFQ (label free quantification) values. Data were analyzed using Perseus (v.1.6.10.50).

For manuscripts utilizing custom algorithms or software that are central to the research but not yet described in published literature, software must be made available to editors and reviewers. We strongly encourage code deposition in a community repository (e.g. GitHub). See the Nature Portfolio guidelines for submitting code & software for further information.

Data

Policy information about availability of data

All manuscripts must include a data availability statement. This statement should provide the following information, where applicable:

- Accession codes, unique identifiers, or web links for publicly available datasets
- A description of any restrictions on data availability
- For clinical datasets or third party data, please ensure that the statement adheres to our policy

Atomic coordinates and EM maps have been deposited in the Protein Data Bank and Electron Microscopy Data Bank under the following accession codes: State A (EMDB-26966, PDB 8CSP), State B (EMD-26967, PDB 8CSQ), State C (EMD-26968, PDB 8CSR), State D (EMD-26969, PDB 8CSS), State E (EMD-26970, PDB 8CST), State C* (EMD-26971, PDB 8CSU), State 1 (EMD-27249, PDB 8D8J), State 2 (EMD-27250, PDB 8D8K), State 3 (EMD-27251, PDB 8D8L). Raw cryo-EM micrographs for each state have been deposited to EMPIAR (EMPIAR-11313).
Data used but not generated in this study: Starting models for model building: human mtSSU-IF3 (PDB 6RW4), human MCAT (PDB 2C2N), human TFB1M (PDB 6AAX), yeast mitoribosome (PDB 5MRC). Search database for IP-MS/MS experiments: Uniprot protein database (2019). Antibodies used during cell line generation: nanobody against E. coli BtuF (PDB 5OVW), nanobody against HIVp24 (PDB 5O2U).

Human research participants

Policy information about studies involving human research participants and Sex and Gender in Research.

Reporting on sex and gender	N/A
Population characteristics	N/A
Recruitment	N/A
Ethics oversight	N/A

Note that full information on the approval of the study protocol must also be provided in the manuscript.

Field-specific reporting

Please select the one below that is the best fit for your research. If you are not sure, read the appropriate sections before making your selection.

☒ Life sciences ☐ Behavioural & social sciences ☐ Ecological, evolutionary & environmental sciences

For a reference copy of the document with all sections, see nature.com/documents/nr-reporting-summary-flat.pdf

Life sciences study design

All studies must disclose on these points even when the disclosure is negative.

Sample size	Sample sizes for cryo-EM datasets were not predetermined. 47,037 (Human dataset), 31,995 (Yeast Ccm1 dataset), and 14,111 (Yeast Rsm22 dataset) micrographs were collected. The number of particles extracted from these micrographs were not predetermined. For comparative IP-MS/MS experiments, one yeast strain was used for three separate purification and mass spectrometry experiments (biological triplicate).
Data exclusions	Mis-aligned, damaged, or contaminating particles were excluded from final reconstructions during image processing. For comparative IP-MS/MS experiments, data were filtered such that a protein must be present in all 3 replicates for at least 1 condition.
Replication	Immunoprecipitation experiments for complex purification and structural analysis were repeated at least 3 times with similar results. Data for comparative IP-MS/MS experiments were gathered from three purification experiments, and three out of three experiments showed similar results. Western blotting experiments were performed three times with similar results for all experiments. Northern blotting experiments were performed twice with similar results.
Randomization	During refinement, the gold-standard approach was used to randomly assign particles to half-sets of data that are independently averaged and compared to obtain resolution estimates.
Blinding	During single particle analysis, particles are randomly assigned into half-sets, thus no blinding is applicable.

Reporting for specific materials, systems and methods

We require information from authors about some types of materials, experimental systems and methods used in many studies. Here, indicate whether each material, system or method listed is relevant to your study. If you are not sure if a list item applies to your research, read the appropriate section before selecting a response.

Materials & experimental systems

n/a	Involved in the study
☐	☒ Antibodies
☐	☒ Eukaryotic cell lines
☒	☐ Palaeontology and archaeology
☒	☐ Animals and other organisms
☒	☐ Clinical data
☒	☐ Dual use research of concern

Methods

n/a	Involved in the study
☒	☐ ChIP-seq
☐	☒ Flow cytometry
☒	☐ MRI-based neuroimaging

Antibodies

Antibodies used: MT-CO1 (Thermo Fisher Scientific, Cat. #459600, Clone 1D6E1A8), MT-ND1 (Thermo Fisher Scientific, Cat. #MA5-42939, Clone 5J5C8), Beta actin (Thermo Fisher Scientific, Cat. #PA1-183), HRP-conjugated goat anti-rabbit IgG (Jackson ImmunoResearch, Cat. #111-035-003), HRP-conjugated goat anti-mouse IgG (Jackson ImmunoResearch, Cat. #115-035-003).

Validation: All antibodies used are commercially available and validated by the manufacturer. For MT-CO1, the antibody was verified by cell treatment to ensure that the antibody binds to the antigen stated.

Eukaryotic cell lines

Policy information about cell lines and Sex and Gender in Research

Cell line source(s): FreeStyle 293-F cells were obtained from ThermoFisher Scientific.

Authentication: FreeStyle 293-F cells were purchased from ThermoFisher Scientific are specially adapted to grow in suspension culture in FreeStyle 293 expression medium. Cells were grown accordingly and matched producer specifications. Cells were not otherwise validated.

Mycoplasma contamination: Cell lines were not tested for mycoplasma.

Commonly misidentified lines (See ICLAC register): No commonly misidentified lines were used in the study.

Flow Cytometry

Plots

Confirm that:

☒ The axis labels state the marker and fluorochrome used (e.g. CD4-FITC).

☒ The axis scales are clearly visible. Include numbers along axes only for bottom left plot of group (a 'group' is an analysis of identical markers).

☒ All plots are contour plots with outliers or pseudocolor plots.

☒ A numerical value for number of cells or percentage (with statistics) is provided.

Methodology

Sample preparation: Cells were washed in 1X PBS with 0.1% BSA before incubation with fluorescent nanobodies corresponding to cell surface epitopes expressed on transfected cells. Cells were subsequently washed and resuspended in 1X PBS with 0.1% BSA before filtering to remove cell clumps and sorting via FACS.

Instrument: BD FACSAria cell sorter (BD Biosciences)

Software: FACSDiva (BD Biosciences)

Cell population abundance: The final population of sorted cells was 0.6% of the starting population. These cells were subsequently analyzed using PCR to confirm genomic integration of tags.

Gating strategy: Cells were first sorted to identify living cells using DAPI staining and isolation of FSC/SSC singlets before sorting for GFP positive cells displaying higher fluorescence than the majority of the cell population. Subsequently, these cells were sorted based on the presence of fluorescence from both fluorophore conjugated nanobodies binding to the cell surface.

☒ Tick this box to confirm that a figure exemplifying the gating strategy is provided in the Supplementary Information.
